# Exploration of recent advancements of nanoparticle-based therapeutics emphasis on diabetic-related chronic wound management: a comprehensive review

**DOI:** 10.1007/s12272-025-01585-7

**Published:** 2025-12-05

**Authors:** Esraa M. Elshazly, Mona G. Arafa, Samia A. Nour

**Affiliations:** 1 Department of Pharmaceutics and Pharmaceutical Technology, Faculty of Pharmacy, The British University in Egypt, Cairo, Egypt; 2 Department of Pharmaceutics and Industrial Pharmacy, Faculty of Pharmacy, Cairo university, Cairo, Egypt; 3 Nanotechnology Research Center, The British University in Egypt, Cairo, Egypt; 4 Chemotherapeutic Unit, Mansoura University Hospitals, Mansoura, Egypt

**Keywords:** Diabetic chronic wound healing, Nanoparticles, Solid lipid nanoparticles, Thermo-responsive gel, Scaling up feasibility, Regulatory approach, Preclinical and clinical studies

## Abstract

The skin is frequently subjected to injuries and disorders encompassing both acute and chronic wounds. Chronic wounds, including diabetic wounds, pose significant clinical problems due to prolonged and ineffective healing processes. Traditional therapies are associated with many limitations. In this regard, nanoparticles (NPs)-based drug delivery systems have emerged as promising solutions for improving chronic wound healing and to overcome the drawbacks of conventional approaches. Furthermore, the functionalization of these NPs through surface modification can increase the overall therapeutic performance. Incorporating them into advanced dosage form maximizes the therapeutic impact. Although their therapeutic promise is high, clinical translation of nanoparticles is hindered by challenges such as manufacturing problems with scaling up production of lipid nanoparticles and the regulatory difficulties related to nanoparticle characterization, such as compliance with FDA criteria for size variation. The current review endeavored to explore the most recently developed nanotechnology-based therapeutic agents that are used in diabetic chronic wound healing, especially SLNs. It also discusses the various surface modification strategies that can enhance therapeutic effectiveness. Further, to maximize the overall efficacy of the drug delivery system and to improve wound healing outcomes, the incorporation of NPs into advanced dosage forms such as thermoresponsive gels has a huge impact. This review also serves as a database for the methodology of collecting the required data, screening, and selection in addition to the pathways from NPs preclinical studies to the stages of clinical approval; moreover, NPs manufacturing and scaling-up feasibility.

**Graphical abstract:**

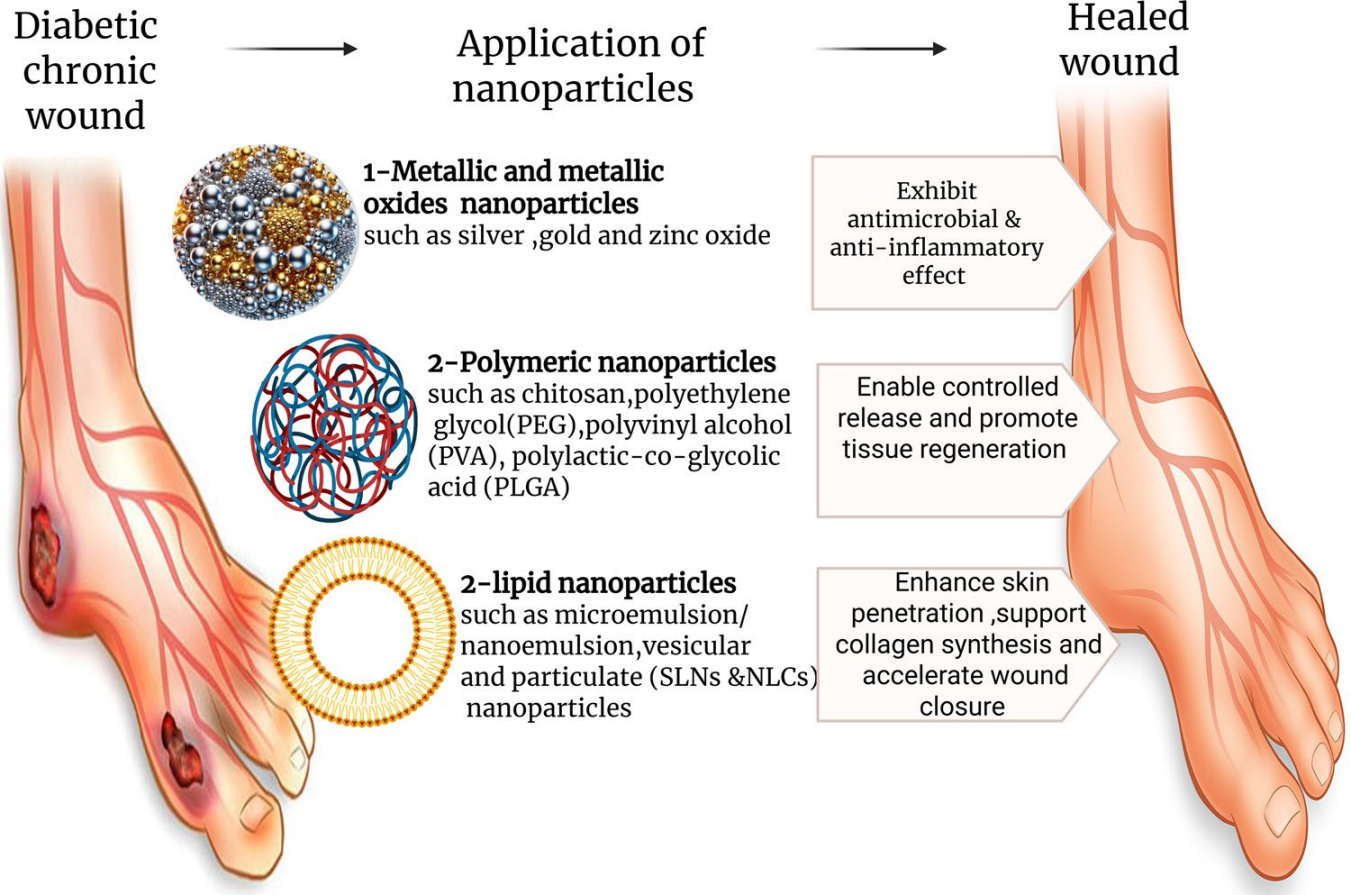

## Introduction

Wound healing in healthy individuals is considered a very accurate and well-organized process. It is a complex series of actions to restore the injury in a timely and orderly manner. This process is composed of three successive and overlapping steps which are hemostasis/inflammatory, proliferative, and remodeling phases (Yasukawa et al. 2020). Otherwise, any impairment in this systematic process can lead to ulcers, scars and serious infections, which delay the healing process and may result in forming chronic wounds. These chronic wounds pose diverse risks and complications including infections, prolonged healing time and tissue damage that can give rise to amputation. One of the leading causes of chronic wounds is diabetes mellitus (DM) (Barnum et al. 2020). The prevalence of DM is increasing worldwide, rendering it the most common chronic disease (Arroyave et al. 2020). This disease is accomplished by many long-term complications, among them, one that happens in foot. Around fifteen percent among all diabetic patients develop foot ulcers (Nandanwar et al. 2021). The etiology is due to many reasons: neuropathy, dysfunctional metabolism of proteins and fats, immune system compromise, and peripheral vascular disease, resulting in loss of sensation in the foot, which leads to an increased risk of repetitive injuries to the patient, ultimately, the formation of ulcers (Aldana et al. 2022). These ulcers are predisposed to infections that cause a delay in the normal healing process, consequently causing major side effects including gangrene and eventual loss of foot. Management of diabetic wounds, especially foot wounds, is very critical. According to the International Working Group on Diabetic Foot (IWGDF) 2019 guidelines on the diagnosis and treatment of foot infection in persons with diabetes, a systemic broad-spectrum antibiotic should be used empirically to avoid any deterioration. Many classes of antibiotic are recommended such as penicillin, fluoroquinolones, cephalosporins, carbapenems, or vancomycin (Lipsky et al. 2020). Fluoroquinolones are a class of broad-spectrum antibiotics active against gram positive and gram-negative bacteria including Methicillin-resistant Staphylococcus aureus (MRSA). Additionally, they have a good tissue penetration, thus, they are advantaged to be used topically or transdermally (Gyssens et al. 2011). Conventional formulations currently available in the market offer numerous potential benefits. However, the oral route suffers from large variations in drug absorption, first-pass effect, and is not possible for unconscious people, unsuitable for individuals experiencing vomiting, has a gradual start of action, and stomach acid and/or digestive enzymes may break down the medication. Therefore, using larger doses of drugs subsequently has greater potential side effects. Furthermore, the intravenous (IV) route has many disadvantages, as it is a short-term solution and an invasive method that requires visiting clinical settings. Hence, the topical and transdermal routes of administration have various advantages over other forms of administered drugs. For example, applying small doses at the site of action, easier administration, reduction in the systemic side effects, and overcoming the frequency of dosing. In addition, topical and transdermal forms are easily administered, painless, convenient, noninvasive, and low in cost. They provide straightforward access to the desired location and prevent systemic negative consequences. However, they have a variety of drawbacks; for example, topical enzymatic activity may lessen the medication’s efficacy, localized rashes may occur on the skin, and limited absorption can lead to deficient bioavailability (Naman et al. 2019). Conversely, the traditional topical antibiotics used nowadays, namely polypeptide antibiotics (bacitracin), aminoglycoside (gentamicin) and fluoroquinolones fail to achieve the required clinical outcomes. Nanotechnology is a highly potential strategy to overcome the above-mentioned problems through the incorporation of the drugs into different advanced nanocarrier systems, in addition to delivering these drugs to the target site, thereby improving the therapeutic efficiency. Thus, using nanoparticles (NPs) is an optimum option for effective chronic wound healing. Many NPs have been reported to promote wound healing such as metallic, polymeric and lipid based NPs (Rajendran et al. 2018; Mihai et al. 2019; Ezhilarasu et al. 2020). Solid lipid nanoparticles (SLNs) have emerged as promising choice for their higher biocompatibility & biodegradability, minimal immunogenicity, also they have natural therapeutic effect on advanced wound healing (Yaşayan et al. 2023). In addition to their great contribution in green synthesis and eco-friendly nanotechnology. Furthermore, to maximize the efficacy of nanocarriers, different approaches must be applied a) incorporation into suitable dosage forms. Most used conventional dosage forms are gels and different dressings. However, in recent years other advanced dosage forms such as thermoresponsive gels have gained momentum, b) coating NPs enhances the wound healing process using polymers or other additives such as chitosan (CS) which consider one of the most used polymeric agents for functionalization of NPs, as it facilitates the migration of neutrophils and macrophages to the wound site, allowing re-epithelialization, decreases the scar tissues (Abosabaa et al. 2020; Cai and Li 2020) and acts as antimicrobial agent (Dai et al. 2011). While the preclinical efficacy of nanoparticles incorporated into gels has been established, their progression towards clinical practice is confronted with enormous regulatory challenges, including the need for thorough nanoparticle clearance profiling and batch-to-batch consistency guarantee (Ghosh et al. 2024) that will be highlighted in the current review. This work also delves into critical analysis of emerging NPs-based approaches for advanced management of diabetic chronic wounds. Particularly focused on engineered SLNs. It explores their methods of preparation, surface coating and incorporation into advanced dosage forms such as thermoresponsive gel. It also highlights the potential to overcome the disadvantages of conventional treatment.

## Review methodology

**Fig. 1:**
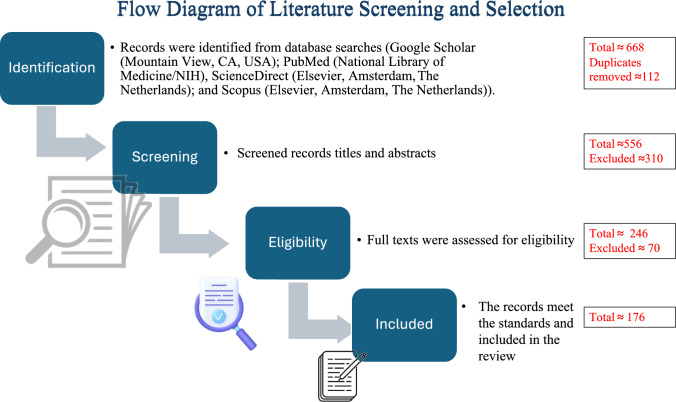
Literature screening and selection flowchart illustrating the identification, screening, eligibility assessment, and inclusion process (Sandoval et al. 2022; Klavsen and Rasmussen 2025) applied to the studies investigating solid lipid nanoparticles and thermos-responsive gels in treating diabetic and chronic wound (2018–2025). Created with Microsoft PowerPoint. The numbers given in the figure are approximate figures from the literature screening process

A comprehensive literature search was conducted, to identify existing research on nanoparticle-mediated therapeutic systems for diabetic chronic wound healing, particularly solid lipid nanoparticles (SLNs) and thermoresponsive gels. To enable methodological transparency and reproducibility, the review was designed and developed according to PRISMA 2020 guidelines (Sylvester et al. 2020; Amaral et al. 2025). Electronic searches were conducted in Google Scholar (Mountain View, CA, USA); PubMed (National Library of Medicine/NIH), ScienceDirect (Elsevier, Amsterdam, The Netherlands); and Scopus (Elsevier, Amsterdam, The Netherlands). The literature search covered research published from January 2018 to August 2025, with a priority for the most recent research from 2020 to 2025. The keywords were searched using truncation and Boolean operators as follows: (“solid lipid nanoparticle* or “nanoparticle* or “nanotechnology”) and (“chitosan coating” or “hyaluronic acid” or “thermoresponsive gel” or “chronic wound healing” opr “diabetic wound”). The literature search was restricted to English-language, peer-reviewed research only (Izzah Md Fadilah et al. 2022). The initial finding yielded approximately 668 items. After the duplicates were removed and inclusion criteria were applied, 176 studies were selected for qualitative synthesis. The studies were required to have the following features to be included: (i) employed solid lipid nanoparticles (SLNs) or thermoresponsive polymeric gels; (ii) measured wound-healing, antimicrobial, or tissue-regeneration endpoints through in vitro or in vivo study; (iii) published from 2018 to 2025; and (iv) full text available in English. In contrast excluded studies were: (i) contained conference abstracts, book chapters, or editorials without unique data; (ii) lacked diabetic wound or wound-healing models; (iii) involved systems other than gel or nanoparticle-based systems; (iv) lacked quantitative wound-healing outcomes or a clearly defined experimental design; (v) or were not available in English or not openly accessible in full text. Moreover, Mendeley Reference Manager was utilized to organize all bibliographic information and research factors. In addition, in vivo preclinical (animal) studies at level I, in vitro mechanistic or formulation studies were ranked at level II evidence, and level III – Clinical/Observational. An overview of the entire literature-selection process contained in (Fig. 1) and (Table 1) details the specific search parameters.

**Table 1:** Overview of the evidence levels for the studies included

Evidence level	Type of investigation	Approximate proportion (%)	Findings
Level I – i*n vivo* (Preclinical)	Animal studies evaluating nanoparticle-based or thermoresponsive formulations	~ 50–60%	Healing and wound closure, histopathology analysis, inflammatory markers assessment, reduction of microbial burden
Level II – in vitro (mechanistic)	Cell culture or mechanistic assays	~ 35–40%	Cytocompatibility, antibacterial activity, cell migration and proliferation
Level III – Clinical/Observational	Human/pilot investigations	< 10%	Healing rate, infection control, safety endpoint
Total	–	100%	–

## Pathophysiology of chronic wounds with a critical focus on diabetic foot ulcer (DFUs)

**Fig. 2:**
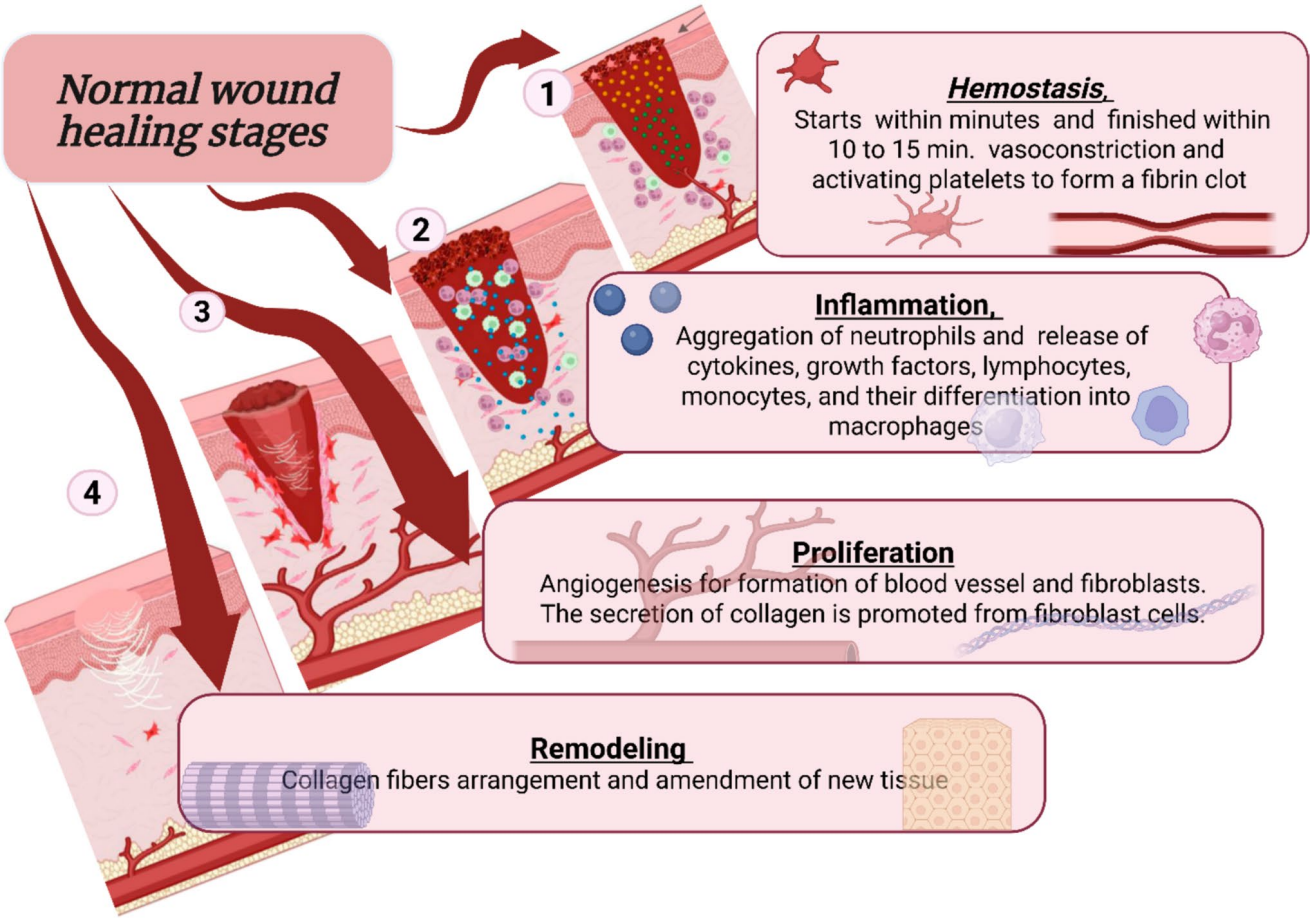
Comprehensive overview of the four stages of normal wound healing: Hemostasis, Inflammation, Proliferation, and Remodeling. (1) Hemostasis consists of active vasoconstriction and platelet activation (Han 2023). (2) Inflammation results from neutrophil migration to the wound, laying down cytokines, growth factors, lymphocytes, and macrophages (Wang et al. 2023a; Zulkefli et al. 2023). (3) Proliferation is characterized by angiogenesis, as well as fibroblast division and collagen deposition. (4) The final phase, remodeling, involves reorganization and cross-linking of collagen fibers (Palanisamy et al. 2022; Tehrany et al. 2023). Created with BioRender.com

**Fig. 3:**
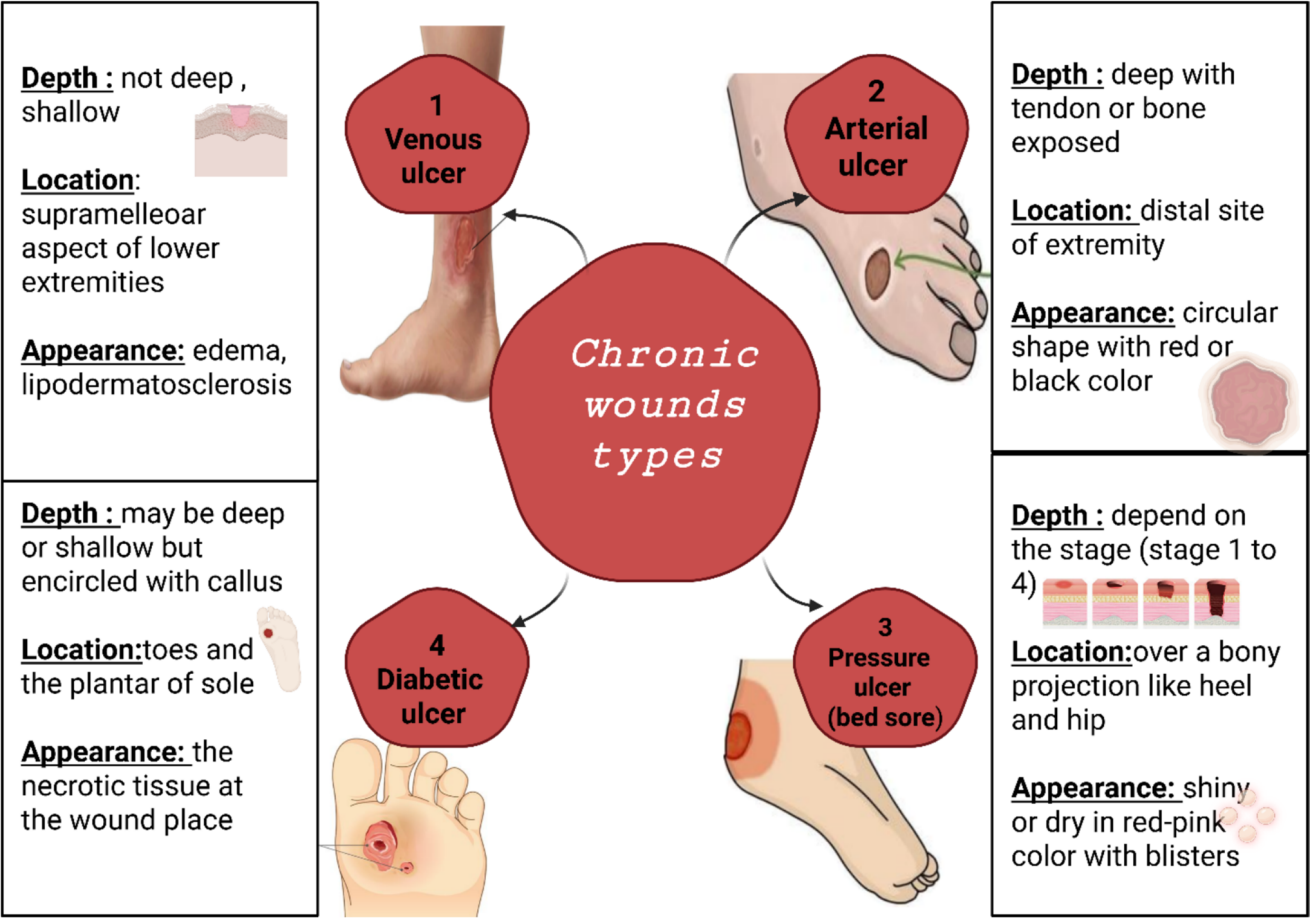
Different types of chronic wounds according to depth, location and appearance. (1) Venous ulcers are usually shallow ulcers in the supramalleolar area of the lower legs, frequently with edema and lipodermatosclerosis. (2) Arterial ulcers are deep, often with visible tendons or bone, and tend to be found in distal areas of the extremities; they are usually circular and red or black. (3) Pressure ulcers, or bed sores, are of differing depth according to stage (1 to 4) and tend to occur over bony prominences such as hips or heels; they are often shiny or dry in appearance, red-pink color, and can be blistered (Kottner et al. 2020; Karahan et al. 2022). Diabetic ulcers range from superficial to deep, are typically covered with callus tissue, and are most likely to be on the toes or on the plantar surface of the foot; diabetic ulcers often contain necrotic tissue at the wound (Falanga et al. 2022). Created with BioRender.com

The Stages of normal wound healing are a well-orchestrated cascade that begins with injury and ends with successful closure to restore skin coherence, (Fig. 2) elucidates the four distinct phases of wound healing process in healthy individuals, which are hemostasis, inflammation, proliferation (angiogenesis, granulation, re-epithelialization) and remodeling (extracellular matrix modeling) phases. The initial phase, hemostasis, starts instantly within minutes when the damage occurs and finished within 10 to 15 min (Han 2023) to stop blood loss by vasoconstriction and activating platelets to form a fibrin clot (Criollo-Mendoza et al. 2023). Furthermore, the formation of blood coagulation helps in stopping the bacterial growth (Canchy et al. 2023). Followed by the second phase in which the inflammation starts with aggregation of neutrophils and release of cytokines, growth factors, infiltration of lymphocytes, monocytes, and their differentiation into macrophages (Wang et al. 2023a; Zulkefli et al. 2023). The third phase is proliferation, it involves angiogenesis, granulation tissues formation and fibroblasts recruitment which prompt secretion of collagen. Lastly the remodeling step that is characterized by collagen fibers arrangement and amendment of new tissue (Palanisamy et al. 2022; Tehrany et al. 2023). On the contrary, the chronic wound does not proceed through a typical, timely, and organized repair sequence as in normal healing. The healing rate of some wounds takes a long time up to three months (Bowers and Franco 2020) while other might not fully healed (Xu et al. 2020). Insufficient healing may occur for many reasons, such as trauma, burns, infection, skin cancer, or underlying medical conditions including neuropathy, diabetes, and immunological factors. Chronic wounds are categorized into pressure ulcers, diabetic ulcers, venous ulcers and arterial insufficiency ulcers. These four primary categories exhibit distinct characteristics, for example, variation in their anatomical location (specifically in the foot regions), depth and visual appearance (Fig. 3) (Bowers and Franco 2020). The most common type, venous ulcers, are typically shallow and located in the supramalleolar aspect of lower extremities (proximal to the ankle). While, arterial ulcers, unlike venous ones, are often deep with exposing tendons or bones. They are located at the distal site of the extremities and characterized by their circular shape with red or black color. The third category comprises pressure ulcers, alternatively referred to as bedsores or decubitus ulcers. They are localized injuries developed on bony projections such as heel, hip and coccyx and they appear shiny or dry with red-pink color, with blisters. They are classified into four stages depending on the severity. Stage I is characterized by superficial tissue damage, while the most severe “stage IV” involves extensive tissue loss extended to the muscle or bone (Kottner et al. 2020; Karahan et al. 2022). Finally, the last category, diabetic ulcer, is the predominant for lower extremities amputation (Falanga et al. 2022). These ulcers primarily developed on the toes and the sole planter. They often show necrotic tissues at the wound site. While they may be deep or show a hallmark feature which is the frequent presence of calluses around the ulcer’s edges. The priority attention of diabetic ulcer care is paramount because DFUs represent severe complication of diabetes mellitus, leading to significant health risk, higher mortality and economic burden. Globally, they are the primary cause of non-traumatic lower limb amputation. These ulcers arise from a complex interaction of metabolic, vascular and neuropathic impairment. Diabetic patients have weakened immune responses that increase the risk of infections and gangrene, necessitating prolonged hospitalization (Kumar et al. 2020). High blood sugar levels that characterize diabetes drive the accumulation of reactive oxygen species (ROS) leading to impairment in blood circulation that resulted in endothelial, microvascular and peripheral nerves damage (Kim 2023). Moreover, polyneuropathy reduces pain sensation, allowing unrecognized wounds to develop into deep ulcers formation. Beyond physiological factors, DFUs are worsen by biofilm forming bacteria like *Staphylococcus aureus* and *Pseudomonas aeruginosa* that promote antibiotic drug resistance and prolonged healing process.

## Conventional treatment of DFUs

The Wagner classification system classifies DFUs into six grades, from 0 to 5, depending upon the severity. They range from grade zero, which is the least severe ulcer to grade five with gangrene of more than two-third of foot (Mohsin et al. 2024). The ulcers should be assessed before initiating the treatment plan followed by the IWGDF 2023 guidelines. The core treatment strategies include wound debridement, wound dressing, infection control, offloading, glycemic control and amputation (Wang et al. 2022b). In case of mild infection, topical antibiotics such as fusidic acid can be used, and in case of severe infection, systemic antibiotics are used. The first line options of such antibiotic are Cephalexin, amoxicillin/clavulanate, clindamycin or fluoroquinolones. While, Vancomycin, linezolid are better option in case of MRSA (Peters et al. 2020). Nevertheless, they have limitations, including frequent dosing, high adverse effects, longer hospitalization, poor drug bioavailability, low patient compliance, impaired infection control, delayed wound healing, and secondary tissue damage while removing conventional dressings (Gorain et al. 2022). In severe uncontrollable cases, these drawbacks may even lead to amputation and mortality in some cases. Therefore, the development of advanced strategies is critical to overcome the disadvantages of conventional therapies to improve healing outcomes and enhance overall therapeutic efficacy.

## Nanotechnology approaches for treating DFUs

Nanotechnology offers a promising strategy to overcome the shortcomings of conventional diabetic foot ulcer treatments. The physicochemical properties of nanomaterials, with their tunable architecture and high surface-area-to-volume ratio, render them improved drug carriers with increased loading efficiency, stability, and bioavailability (Cheng et al. 2023). Nanomaterials are generally formulated as targeted, sustained, and controlled release delivery systems, which greatly facilitate tissue regeneration and wound healing mechanisms (Ullah et al. 2025; Banerjee et al. 2025). Wound healing under diabetic conditions is optimal if tissue regeneration is stimulated and infection prevented. Thus, nanocarrier systems accomplish dual actions: regenerative activities (e.g., hyaluronic acid-induced angiogenesis or immunomodulation by chitosan induce re-epithelialization and collagen synthesis), while antimicrobial activities (e.g., reactive oxygen species generation by metal nanoparticles or bacterial membrane disruption by chitosan) prevent chronic infection. This synergistic effect is supported by new evidence from next-generation platforms, including 3D-printed quaternized chitosan nanoparticles loaded with mupirocin, which show strong antibacterial activity along with improved skin regeneration (Almajidi et al. 2024). Collectively, these reports provide evidence for the clinical utility of multifunctional nanoplatforms that combine antibacterial and regenerative functions for improved diabetic foot ulcer therapy. Various forms of nanoparticles, mainly inorganic NPs (metallic, metallic oxide and nanobioceramics) and organic NPs (lipid and polymeric), have been found to hold potential as therapeutic agents for the therapy of chronic, non-healing diabetic ulcers (Gowda et al. 2023). Besides, coating nanoparticles with biocompatible polymers such as chitosan enhances their bioavailability and interaction with cells, also improves targeting capabilities, promotes delivery effectiveness, increases cellular uptake and penetration, additionally, aid in controlled drug release (Wang and Mattoussi 2020; Malabanan et al. 2023). Moreover, these surface modified NPs improve stability during delivery through preventing aggregation and increasing circulation time, this means ensuring better accumulation at the target sites (Burki et al. 2023). Among various coating techniques, electrostatic interaction is widely utilized, it is an interaction between opposite charges molecules. One of the most positively charged polymers used in surface coating is chitosan (CS) which is a natural polysaccharide derived from the partial deacetylation of chitin, typically found in the exoskeleton of crustacean and in fungal cell walls. CS is nontoxic, harmless, relatively stable and biocompatible with organs, tissues and cells, making it valuable in various fields particularly in wound healing (Jiménez-Gómez and Cecilia 2020; Desai et al. 2023). Additionally it serves as hemostatic agent to facilitate blood clot (Aldayel et al. 2023) and it acts as antimicrobial agent due to carrying positively charged molecules that interact with the negatively charged bacterial membrane (Arafa et al. 2023). This interaction disrupt the bacterial membrane, causing leakage of the intracellular constituent and eventually bacterial death (Feng et al. 2021). Aside from its antimicrobial and hemostatic effects, CS also exhibits immunomodulatory activity, stimulating anti-inflammatory cytokines such as interleukin-10 (IL-10) to promote tissue regeneration following the induction of macrophages, and the increased release of pro-inflammatory cytokines during the initial phase of wound healing. In addition, CS NPs reduce excessive tumor necrosis factor-alpha (TNF-α) and interleukin-6 (IL-6) during the inflammatory stage while enhancing macrophage recruitment and partial M1 (pro-inflammatory) activation. Subsequently, CS facilitates the conversion of M1 macrophages into the M2 (anti-inflammatory/pro-healing) phenotype, which are characterized by increased interleukin-10 (IL-10) and transforming growth factor beta (TGF-β), alongside reduced oxidative stress, facilitated by chitosan’s cationic amino groups. This balanced modulation of the immune system accelerates the process of moving from the inflammatory to the proliferative phase of wound healing (Ghattas et al. 2025). Another commonly used polymer is hyaluronic acid (HA), which creates a multifunctional platform that improves drug delivery, especially for skin application and therapeutic utility (Buckley et al. 2022). Hyaluronic Acid (HA), similar to chitosan contributes to immunomodulation by recruiting neutrophils and macrophages, modulating inflammation, and creating a pro-healing environment (Rosales et al. 2024). In addition, it modulates cell migration and angiogenesis, which play a crucial role in wound repair (Martin et al. 2024) as it binds to receptors CD44 (cluster of differentiation-44) and RHAMM (receptor for hyaluronan-mediated motility) on endothelial cells to promote VEGF (vascular endothelial growth factor) and FGF (fibroblast growth factor), activating signaling pathways that lead towards new vessel formation (Elvitigala et al. 2024). Numerous other polymers and molecules are also employed in functionalization such as Poly vinyl pyrrolidone (PVP) and sodium alginate (Şanli et al. 2008; Afriani and Sutanti Budikania 2020; Afriani et al. 2024).

### Nanoparticles based approaches for DFUs management

#### Metallic, metal oxides NPs and nanobioceramics

Metallic nanoparticles including silver and gold are ideal candidates for DFUs healing due to their distinctive antimicrobial, stability, safety, regenerative properties and high surface to volume ratio. Metallic oxide (such as zinc oxide) exhibits high anti-inflammatory effects and tissue regenerative property (Faghani and Azarniya 2024). The reason behind their antibacterial effect is their potential to produce reactive oxygen species (ROS) which can kill bacteria. Also, they can bind to RNA and DNA which further hinder the bacterial replication (Vijayakumar et al. 2019). Furthermore, green synthesis of such metallic NPs improves their safety for application in biological tissues, in addition it is considered a cost effective and ecofriendly method (Salem and Fouda 2021; Dikshit et al. 2021). These materials are recognized for their ability to reduce bacterial load while also contributing to immunomodulation and angiogenesis, a key part of the proliferative phase of healing by upregulating factors like VEGF (Vascular Endothelial Growth Factor) and FGF-2 (Fibroblast Growth Factor-2). The distinct mechanisms employed by each nano-type to achieve this effect will be discussed in detail (Carton and Malatesta 2024; Ramachandran et al. 2024). In addition to metals and metal oxides, nanobioceramics (silica nanoparticles, bioactive glass, and hydroxyapatite) represent a further inorganic platform for the treatment of wounds. Unlike metallic nanoparticles, their mechanism mostly entails controlled drug delivery of therapeutic ions (e.g., Ca^2^⁺, Si^4^⁺, Zn^2^⁺), with sustained activity at the wound bed, promotion of angiogenesis, control over collagen deposition, and modulation of inflammatory processes. Recent studies indicate that nanobioceramics are capable of promoting tissue regeneration and the long-term delivery, offering a new way to regulate the healing process of chronic wounds (Al-Naymi et al. 2024). Despite their promising properties in treating chronic wounds, they have limitations such as cytotoxicity and genotoxicity. In addition, they can cause oxidative damage to cells based on particle size, dosage, and duration of exposure while, simultaneously, exerting antimicrobial, antioxidant, and regenerative activities in wound healing. In animal models such as Danio rerio (Mahjoubian et al. 2023), repeated exposure to silver- and zinc oxide nanoparticles increased lipid peroxidation and DNA damage indicators; likewise, titanium dioxide nanoparticles have also been shown to penetrate impaired skin and induce oxidative stress, apoptosis, and cytoskeletal disruption (Pormohammad et al. 2021). These highlight the priority of dose optimization, safe synthesis/coating and in vivo stringent safety evaluation before translating these materials to the clinic (Sheikh-Oleslami et al. 2023; Medic et al. 2024). It is worth mentioning that additional factors influencing the efficacy of metallic, metal oxides and ceramics NPs include their concentration, duration of exposure, and particle size. For instance, many reports have stated that optimal cytocompatibility of Ceramics NPs has been observed at 150–800 µg/mL. However, extended contact or excessive dosing beyond this range can increase oxidative stress and compromise cell viability. Generally, particles smaller than 100 nm demonstrate increased bioactivity and more controlled ion release, though longer exposure times may still lead to oxidative stress (Pajares-Chamorro and Chatzistavrou 2020; de Souza et al. 2024).

##### Silver NPs

Infection is considered one of the leading causes of delaying wound healing. Therefore, Silver nanoparticles (AgNPs) are increasingly valued in DFUs care due to their excellent bacteriostatic bactericidal and antibiofilm activities (Hemamalini et al. 2023). Silver, especially silver nitrate, has been recognized in various marketed products as strong antimicrobial agent, however, AgNPs have superior advantages in having larger surface area and remarkable physicochemical properties which enhance their antimicrobial efficacy (Liao et al. 2019). Consequently, they offer superior contact for interacting with microorganisms compared to traditional silver agents. Their antibacterial mechanism of action relays in releasing free radical which causes oxidative damage and further bacterial destruction. Additionally, AgNPs release Ag⁺ that can interact with the negatively charged bacterial cell membrane, leading to leakage of intracellular content and structural damage. Another reason for AgNPs strong antimicrobial activity is disrupting critical metabolic processes (inactivating DNA synthase and respiratory enzymes) upon entering the bacterial cell. This is because of the interaction with phosphorus and sulfur groups present in the protein components of the microbial plasma membrane and cell wall (Capanema et al. 2023). In addition, their actions against drug-resistant strains in experiments in the laboratory with plant-derived AgNPs encompass the formation of reactive oxygen species (ROS) that induce oxidative damage in bacterial DNA, proteins, and lipids (Laib et al. 2025). As previously mentioned, AgNPs downregulate the inflammatory reaction by blocking pro-inflammatory cytokines such as TNF-α (tumor necrosis factor-alpha), IL-6 (interleukin-6), and IL-1β (interleukin-1 beta) thus creating a well-adjusted inflammatory milieu favorable for healing. Simultaneously, they also stimulate angiogenesis, fibroblast proliferation, and collagen formation, thus contributing to enhanced tissue repair (Ali et al. 2023). As mentioned, the safety profile and the therapeutic performance of AgNPs are affected by their concentration, duration of exposure, and particle size, accordingly, AgNPs are generally found to have good antibacterial effects at low-to-moderate doses of around ≤ 50 µg/mL. However, higher exposures and concentrations ranging from 70 to 100 µg/mL have been associated with excessive ROS production and mitochondrial or oxidative cell injury in experimental models. AgNPs with sizes < 50 nm generally demonstrate increased bioactivity due to higher surface reactivity and more controlled ion release, although longer exposure times may pose cytotoxic risk (Paladini and Pollini 2019; Eker et al. 2024). Through their multifaceted mechanism of actions, AgNPs demonstrate significant antimicrobial against diverse range of pathogens including those with multidrug resistant bacteria. AgNPs can be synthesized through different methods of preparation such as physical, chemical and biological way. Biofabricated AgNPs, formed through utilizing biological or plant extracts, are considered less harmful, cost effective and more biocompatible method (Saddik et al. 2024). Table 2 brings together the therapeutic uses of silver nanoparticles (AgNPs) in chronic wound management, including data on formulations, active ingredients, and reported outcomes. The most pertinent preclinical results are as follows: (1) bare AgNPs embedded in cellulosic nonwoven fabrics were shown to possess strong antibacterial activity, with up to 19 mm inhibition zones against E. coli (Hemamalini et al. 2023); (2) chitosan films with incorporated AgNPs have shown sustained release of silver ions and increased rates of wound closing compared to traditional treatments (Pansara et al. 2020); (3) polyvinyl alcohol hybrid hydrogels effectively inhibited bacterial growth without reducing biocompatibility, though further in vivo validation is needed (Capanema et al. 2023); (4) Azithromycin loaded AgNPs incorporated into Hydroxypropyl methylcellulose (HPMC) gels showed enhanced antimicrobial activity against MRSA and E. coli, as reflected by lower minimum inhibitory concentrations (MICs); and (5) combinations of tannylated calcium peroxide and AgNPs in alginate gels exhibited synergistic antibacterial activity against Gram-positive and Gram-negative bacteria (Bîrcă et al. 2024). Notably, gallocatechin-AgNP gauze patches enhanced collagen synthesis and exhibited antioxidant activities indicative of 80% reduction in oxidative stress faster healing of diabetic wounds. Collectively, these findings underscore the multilateral possibilities of AgNPs as a remedy for oxidative stress, infection prevention, and tissue restoration for chronic wound treatment.

**Table 2:** Summary of AgNPs used in chronic wound treatment: active ingredients, coating, dosage forms and outcomes

Drug/ Active ingredient	Surface coating	Dosage form	Key findings	Reference
AgNPs only	Uncoated	Chitosan film	Sustained release of Ag⁺ and increasing anti-microbial effect (*E. coli)* and Improved wound closure rate compared to other conventional treatment and chitosan film without silver nanoparticle in vivo	(Pansara et al. 2020)
Gallocatechin (green tea)	Uncoated	Cotton gauze patches	In vivo diabetic rats model results: Significant improvement in wound healing, reduction in oxidative stress and inflammation suppression	(Vendidandala et al. 2021)
AgNPs only	Uncoated	Cellulosic nonwoven fabric	In vitro test showed maximum zone of inhibition up to 19 mm that indicate potent antimicrobial properties	(Hemamalini et al. 2023)
AgNPs only	Uncoated	Hybrid hydrogel of PVA	The hydrogel significantly inhibit the bacterial growth and it is safe with good levels of biocompatibility and in vivo given confused results and the researchers suggest further studies on real models	(Capanema et al. 2023)
Tannylated Calcium Peroxide and silver	PVP	Alginate gels	Ag and tannylated Ca peroxide NPs exhibit synergistic antibacterial effect (S. *aureus),* and high inhibitory effect on G- and G + bacteria, in vitro	(Bîrcă et al. 2024)
Azithromycin	Azithromycin adsorbed onto the surface	HPMC gel	High zone of inhibition and strong antimicrobial effect mainly against E. coli and MRSA, in addition to decrease level of MIC with significant antimicrobial activity in vivo study	(Saddik et al. 2024)

*AgNPs* silver nanoparticles, *PVA* polyvinyl alcohol, *PVP* polyvinylpyrrolidone, *HPMC* hydroxypropyl methylcellulose, *MIC* minimum inhibitory concentration, *E. coli Escherichia coli*, *G⁺* gram-positive, *G⁻* gram-negative, *Staphylococcus epidermidis S. aureus*

##### Gold nanoparticle

Gold nanoparticles (AuNPs) have drawn a lot of interest in diabetic wound healing due to unique physicochemical properties, small size, tunable functionalized surface, and multifunctional capabilities. AuNPs accelerate healing through different mechanisms, first through their anti-inflammatory and antioxidant effects as they can scavenge reactive oxygen species (ROS) by restoring redox balance and activating the nuclear factor erythroid 2–related factor 2 (Nrf2) pathway (Kim et al. 2021), in addition to their antimicrobial and proangiogenic effects (Fereig et al. 2022). Regarding immunomodulatory and angiogenic effects, gold NPs proangiogenic activity is related to the upregulated expression of multiple angiogenic factors including VEGF hypoxia-inducible factor-1α (HIF-1α) and fibroblast growth factor (FGF), which are critical for promoting blood vessel formation and eventually extracellular matrix remodeling (Zheng et al. 2019; Shaabani et al. 2021). In a research study, IL-4 (interleukin-4)-functionalized AuNPs decreased oxidative stress and promote macrophage polarization towards the regenerative M2 (macrophages) phenotype, with increased granulation tissue thickness and wound closure in diabetic mice (Wang et al. 2020; Yao et al. 2023). Building on our prior discussion, the therapeutic outcomes of AuNPs are similarly governed by concentration, particle size, and duration of exposure. Several reports have stated that at concentrations of approximately 15–40 µg/mL and particle sizes of about 10–50 nm, they generally promote angiogenesis and inhibit inflammation (Poomrattanangoon and Pissuwan 2024). However, in some cases, higher cumulative exposures have been associated with reduced cell viability and increased cellular distress markers. In general, these data underscore the need for the determination of safe dose exposure size windows, protective surface coatings, controlled-release systems, and standardized in vivo safety testing (Salama et al. 2024; Gounden and Singh 2025). AuNPs are typically synthesized through chemical or green synthesis. An example of a milestones in AuNPs chemical synthesis is Turkevich method (Dong et al. 2020). This method is basically a citrate reduction in the presence of gold precursor Chloroauric acid (HAuCl₄) or sodium tetrachloroaurate (NaAuCl4) (Oliveira et al. 2023). The green synthesis using some plant extract is more safer, easy, ecofriendly and it does not require toxic agents or hazard condition (Jafarizad et al. 2019). Table 3 summarizes recent studies regarding the application of gold nanoparticles in various formulations for diabetic wound healing, including data on surface coatings, formulation types, and key therapeutic outcomes.

**Table 3:** Overview of recent gold nanoparticles studies for diabetic wound management

Drug/active ingredient	Surface coating	Dosage form	Key findings	References
DsiRNA	Chitosan	Thermoresponsive hydrogel	Acceleration of wound healing, enhancement of blood vessel formation and reduction in biofilm formation and bacterial infection, in vitro and in vivo	(Nor Azlan et al. 2021)
*Chamaecostus cuspidatus* extract	Uncoated	Not mentioned	Higher wound closure rate mainly due to the antioxidant effect (In vivo* study*)	(Ponnanikajamideen et al. 2019)
No drug	Chitosan	Hydrogel dressings	Effective eradication of MRSA, reduction in inflammation and promotion in angiogenesis enhancement of collagen deposition and remodeling within the wound site (In vivo study)	(Meng et al. 2024)
pDNA	AMPs	Hydrogel based dressing	Promoting both the antibacterial action and the neocapillaries formation (In vivo study)	(Wang et al. 2022a)
A phytochemical acalyphaindica	Uncoated	Cotton fabric	High antibacterial activity was observed against *E. coli* and *S. aureus*, the free radical scavenging activity was 80%, which referred to high antioxidant activity, increase reepithelialization of the wound surface in a duration of 15 days, in vivo	(Boomi et al. 2020)

*DsiRNA* double-stranded small interfering RNA, *pDNA* plasmid DNA, *MRSA* methicillin-resistant, *AMPs* antimicrobial peptides, *E. coli Escherichia coli*, *Staphylococcus epidermidis S. aureus*

##### Zinc oxide nanocarriers

ZnO NPs possess a range of therapeutic properties, including pro-healing, anti-inflammatory, and selective antibacterial action, rendering them of particular interest to processes of wound healing, particularly within the complicated diabetic chronic wounds context (Singh et al. 2021). Among the prominent features that distinguish ZnO NPs is their potential to function as zinc ion reservoirs with controlled bioactive Zn^2^⁺ ion release. These ions serve as crucial cofactors to various transcription factors and enzymes such as collagenases, DNA polymerases, and matrix metalloproteinases (MMPs) that have crucial roles in regulating fundamental processes, including extracellular matrix remodeling, collagen synthesis, and keratinocyte proliferation (Larijani et al. 2024). Moreover, since Zn^2^⁺ is a micronutrient ingested through the diet, its ions have intrinsic biocompatibility and selectively target infection with little cytotoxicity against host cells. Their anti-inflammatory activities assist in providing a healing-facilitating environment by lowering the chronic inflammation that is typically present in diabetic wounds. Consistent with what has been established for such nano-types, they modulate immunity by suppressing TNF-α, IL-6, and IL-1β, and promote angiogenesis by inducing the expression of VEGF, all of which are responsible for healing diabetic wounds (Hassan et al. 2021; Yin et al. 2022). Notably, at low concentrations, approximately 1–7.5 µg/mL, ZnO NPs have been found to activate fibroblasts and endothelial cells. However, intense exposures of about 35–50 µg/mL have contributed to oxidative stress and genotoxicity in dermal and endothelial cells. Smaller ZnO NPs, usually less than 40 nm, exhibit better antibacterial and wound-healing properties owing to their controlled release of Zn^2^⁺ ions and higher surface reactivity (Singh 2019; Wiesmann et al. 2021). Recent preclinical research, as reviewed in Table 4 has shown the effectiveness of ZnO NPs.

**Table 4:** Summary of current research on ZnO NPs for managing diabetic wounds

Drug/active ingredient	Surface coating	Dosage form	Key findings	References
Only ZnO NPs	Sodium alginate	Cellulosic dressing (bleached cotton fabric)	Effective bactericidal effects. In vivo studies showed a wound decrease of 93.5% over 21 days	(Elsawy et al. 2022)
ZnO-NPs derived from *Althaea officinalis* flowers, marshmallow + chitosan	Chitosan	2% chitosan gel	Anti-inflammatory effect of ZnO and reduction in pro-inflammatory cytokines, in vivo	(Elhabal et al. 2024)
Only ZnO NPs	Uncoated	Thermo-responsive hydrogel	Antimicrobial properties toward (MRSA). High wound-healing effectiveness, by increase collagen synthesis and increased vascularization. In addition, the hydrogel exhibited biocompatibility as well as non-toxicity. in vivo	(Wang et al. 2024)
Only ZnO NPs	Uncoated	Electrospun nanofibrous membrane	The membrane’s pore structure promoted gas change while maintaining the environment moist enough for the wound. Promoted accelerated wound closure as well as increased quality of wound healing, in vivo	(Khan et al. 2021)
ZnO NPs + chitosan + Insulin	Chitosan	Local injection of insulin and nanocomposite membrane	The synergistic effects of local insulin injection and the nanocomposite chitosan/ZnO membrane both cover infection management and structural support. Insulin facilitates angiogenesis and cellular regeneration by stimulating the expression of growth factor, in vitro and in vivo	(Hussein et al. 2022)

All studies employed in vivo study on diabetic rats

*ZnO NPs* zinc oxide nanoparticles, *MRSA* methicillin-resistant, *Staphylococcus aureus*

#### Polymeric nanoparticles

Polymeric nanoparticles, whether natural, synthetic, or composites, offer distinct benefits due to the inherent attributes of constituent materials. They form an efficient treatment approach for chronic wound healing. Chitosan and alginate are examples of natural polymers, exhibiting antibacterial property, gelation ability, together with being biocompatible, degradable, and retaining moisture, attributes in enhancing wound healing. Synthetic polymers, especially polyethylene glycol (PEG) and polylactic acid (PLA), offer structural stability while being degradable, rendering them suitable for repeated application. Composite polymers, including, chitosan-alginate, Polyethylene glycol–polyvinyl alcohol (PEG-PVA), combine the attributes of single units toward increased structural stability, hydration, antibacterial performance. Polymeric particles, thus, provide an efficient, versatile means of treating chronic wounds (Gillella et al. 2024). Recent research studies of polymeric based nanoparticles have shown promising strategies toward managing diabetic wounds. One study proves that asiaticoside-loaded nano spheroids, employing chitosan-PLGA composites prepared by emulsion solvent evaporation processes, significantly increased collagen synthesis and accelerated wound coverage by epithelium in diabetic rodent models upon topical application via matrices of hydrogel (Narisepalli et al. 2023). Also, ionically cross-linked nano-spheroids containing doxycycline exhibited extensive antimicrobial efficiency, as well as increased wound resolution parameters, in topical formulation as topical creams (Hosseini et al. 2019). Moreover, experimental studies utilizing the biphasic emulsion method for PLA nanoparticles synthesis, aimed to encapsulate insulin, exhibited sustained kinetic release profiles. In addition, hydrogel formulations increased the rate of wound contraction in models of diabetes (Ribeiro et al. 2020). Another recent finding involves antioxidant-rich ferulic acid-chitosan nanocomplexes prepared by means of anionic gelation, which, when added to matrices of hydrogel, significantly increased tissue regeneration markers in vivo (Bhardwaj and Jangde 2024). Furthermore, A recent approach involved platelet lysate-encapsulated NPs prepared by solvent evaporation via two-phase emulsion and added to thermoresponsive matrices of hydrogel for targeted factor release. The combinatorial system allows for the promotion of angiogenesis by enabling bioactive substance release, while preclinical trials established increased wound closeup time, coupled with histological return, in chronic models of diabetes (Bernal-Chávez et al. 2020). Despite the advantages of polymeric NPs in chronic wound healing, they have several drawbacks. Their safety profile in humans over the long term is not known, and they are mostly in preclinical stage. Concerns include their potential deposition in organs such as liver, spleen, and kidneys, in addition to cytotoxicity at high doses (Chen et al. 2025). Physical stability is also of concern: some polymer formulations can lose the drug payload, degrade, or aggregate on storage or when exposed to wound fluids (Shalaby et al. 2022). Reduction can be necessary on repeat dosing if drug release rates and site occupancy are inadequate (Nunes et al. 2022). Antimicrobial activity can be achieved for the majority of polymeric nanoparticles by secondary functionalization with antimicrobial agents, a step adding to cost, complexity, and potential regulatory difficulties (Fu et al. 2024).

#### Lipid based nanoparticles in treating chronic wounds

Lipid-based nanoparticles are tiny spheres formed from lipids. They serve as effective carriers for the delivery of pharmaceuticals and therapeutic agents to the target site. Their versatility and unique characteristics highlight their potential to enhance diabetic wound treatment efficacy and improve recovery timelines patient in chronic wound medical settings (Li et al. 2025). Because they are composed of lipids like those in biological membranes, making them highly biocompatible and biodegradable, thus reducing the risk of toxicity and simplifies broken down and safely eliminate from the body (Abosabaa et al. 2020; Gangavarapu et al. 2024). In addition, their lipid nature allows integration with cell membrane to enhance deeper penetration into the wound tissue. The versatility of lipid nanoparticle allows them to incorporate various kinds of drugs and other therapeutic agents either they are hydrophilic or lipophilic. As reported diabetic wounds often require longer time for complete healing, lipid nanoparticles can effectively sustain the drug release by tailored release kinetics, that leads to reducing the frequent dosing. Moreover, they can easily adapt to gels, creams or other topical dosage form, allowing easy formulation and increasing the patient compliance (Motsoene et al. 2023; Ullah et al. 2024). These nanoparticles can be broadly divided into three types: micro/nano emulsion, vesicular systems, and particulate lipid carriers (SLNs, NLCs) (Rehman et al. 2024). Despite the benefits, lipid nanoparticles used in chronic wound healing possess certain drawbacks. Such in case of SLNs that have low residence time at the site of application, limited drug loading capacity, and polymorphic transitions that can detract from long-term stability (Alloush and Demiralp 2025). To maintain therapeutic levels, multiple applications of certain lipid nanoparticle formulations may be required, and they may not penetrate tissue to sufficient depth (de Souza et al. 2020). The other challenges include batch-to-batch variations, limited scalability in production, and surfactant-mediated irritation (Wathoni et al. 2024).

##### Microemulsion and nano emulsion nanoparticles

Microemulsions and nanoemulsions, colloidal dispersion systems, are widely applied in chronic diabetic wound management due to their ability to effectively incorporate both polar and non-polar drugs and enhance their stability, solubility, and bioavailability. Microemulsions are kinetically stable systems, utilize surfactants and cosurfactants while nanoemulsion only utilizes surfactant. Both systems possess increased drug penetration, controlled release rates, and the possibility of delivery to infected or compromised tissues selectively (Chhabra et al. 2023), proving their effectiveness in a variety of applications as reported from recent studies (Table 5).

**Table 5:** Current studies investigate the application of microemulsion/nanoemulsion NPs in the treatment of diabetic wounds, focusing on the key findings about efficacy, and therapeutic potential

Drug/active ingredient	Surface coating		Dosage form	Key findings	References
Collagen peptides	Uncoated		Oral (no specific dosage form)	In vivo (diabetic mice): ~ 95% increase in wound healing area; enhanced cellular repair processes	(Fortuna Rodrigues et al. 2023)
Linezolid	Uncoated		Topical (no specific dosage form)	In vivo (diabetic Sprague–Dawley rats): improved antibiotic efficacy, enhanced wound contraction, better re-epithelialization, and improved histology vs. control	(Haider et al. 2022)
Insulin	Uncoated		Topical (aloe vera gel)	In vitro and in vivo studies: Synergistic effect of aloe vera and insulin combination, increment of the overall wound healing process and enhancement in wound contraction (75% by 15th day) over control	(Chakraborty et al. 2021)
Curcumin and α-Tocopherol (Vit.E)	Uncoated		Topical (no specific dosage form)	In vitro studies & in vivo test on diabetic Sprague–Dawley rats: synergistic antioxidant effect due to combination of ponent antioxidants curcumin and vit. E, synergistic antimicrobial effect as curcumin exhibits antimicrobial action and enhanced stability of curcumin in the nano emulsion, against degradation upon adding vit. E	(Ali et al. 2022)
Levofloxacin		Uncoated	Topical Carbopol 934 gel)	In vitro and in vivo models: Improvement in diabetic wound healing and elevated collagen synthesis, wound contraction, and cell proliferation compared to control groups	(Valizadeh et al. 2021)

*Vit.E* vitamin E

##### Vesicular nanoparticles

Vesicular drug delivery systems (VDDS) are a major achievement in nanotechnology, particularly in the context of chronic wound management (Matei et al. 2021). VDDS employ biocompatible vesicles like liposomes, glycerosomes, and ufasomes to encapsulate therapeutic drugs and thereby enhance the stability, bioavailability, and targeted delivery of drugs. VDDS can boost the efficacy of treatment and reduce side effects through controlled and sustained release of drugs (Rajizadeh et al. 2023). Their ability to promote tissue regeneration, suppress inflammation, and control infection in chronic wounds addresses significant problems in the successful clinical management of these VDDs (Mansouri et al. 2022; Kim et al. 2025). Recent studies of vesicular systems and their application in healing chronic wounds are listed in (Table 6).

**Table 6:** Thorough analysis of current investigation into the role of different vesicular nanoparticles in the treatment of diabetic wounds

Vesicular delivery system	Drug/active ingredient	Surface coating	Dosage form	Key findings	References
Glycerosomes	Resveratrol	Uncoated	2% HPMC Gel	In vitro & In vivo: enhanced resveratrol flux and permeability in comparison to free resveratrol	(Belal et al. 2024)
Cubosomes	ROX	Poloxamer 407	Thermoresponsive hydrogel	Enhancement in wound healing, increasing wound closure by 93%, Improvement in vascularization and collagen deposition due to higher expression of HIF-1α (in vivo study)	(Nasr et al. 2024)
Ufasomes	Rosuvastatin	Chitosan	Chitosan gel	In vitro, ex vivo and in vivo studies: Sustained release of drug, reduction in systemic absorption and remarkable diabetic wound healing	(M El-Masry et al. 2024)
Liposome	SDF-1α gene	HA	Gel	In vivo* results:* enhancing tissue regeneration, increasing collagen deposition and angiogenesis	(Wang et al. 2023b)

*ROX* roxadustat, *HPMC* hydroxypropyl methylcellulose, *HA* hyaluronic acid, *SDF-1α* stromal cell-derived factor-1 alpha

##### Particulate lipid carriers

Solid lipid nanoparticles (SLNs) and nanostructured lipid carriers (NLCs) are prominent particulate lipid carrier classes with widespread applications in wound treatment. (de Souza et al. 2020). Unlike SLNs, which are made up of solid lipids only, NLCs contain both liquid and solid lipids (Viegas et al. 2023). SLNs provide a strong formed matrix characterized by more sustained release of the drug and enhanced drug stability (Souto et al. 2020). Notably, both NLCs and SLNs can maintain constant therapeutic levels of the drug at the wound site, maintaining a prolonged healing process (Wathoni et al. 2024).

**Fig. 4:**
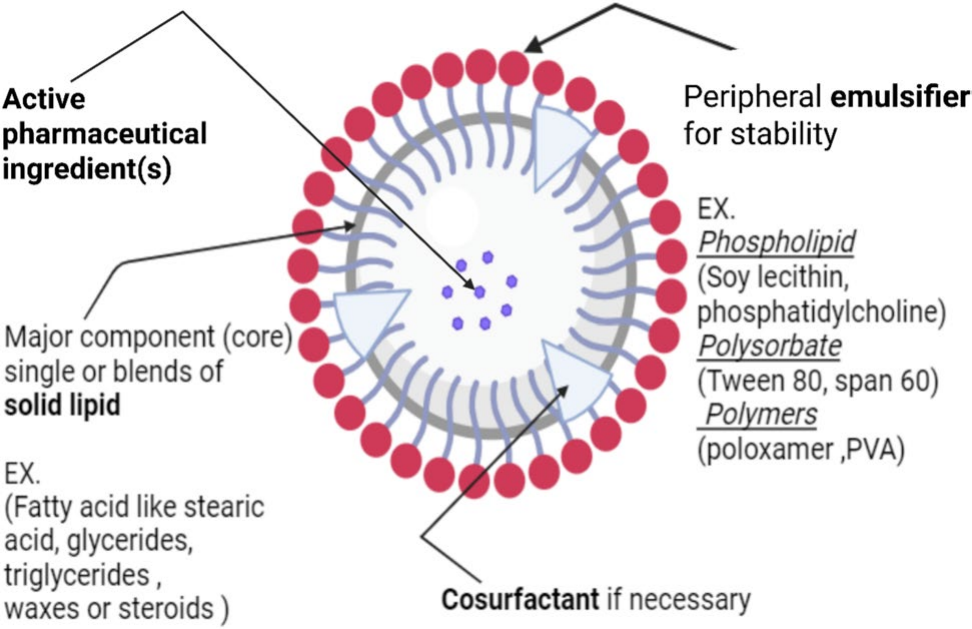
Schematic illustration of the structural components of a solid lipid nanoparticle, which consist of (1) The active pharmaceutical ingredient(s) are placed within the nanoparticle core. (2) The core is predominantly a solid lipid, either one lipid or a mixture of lipids such as fatty acids, glycerides, triglycerides, waxes, or steroids. (3) The structure is stabilized by a surrounding emulsifier, like phospholipids like soy lecithin or phosphatidylcholine, polysorbates like Tween 80 or Span 60, or polymers like Poloxamer or PVA. (4) There is also a co-surfactant within the structure (German-Cortés et al. 2023). Created with BioRender.com

**Fig. 5:**
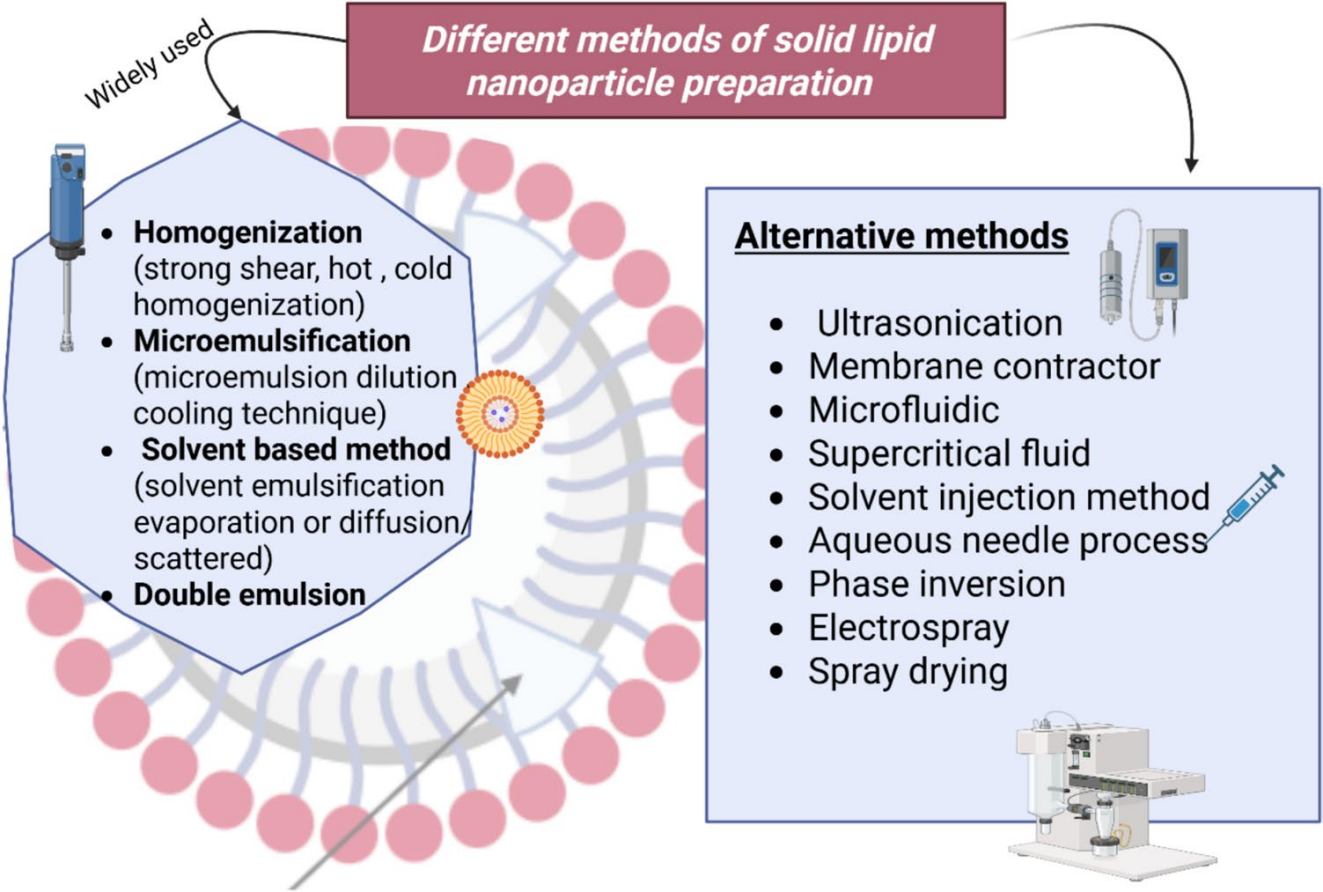
An overview of diverse methods of solid lipid nanoparticles (SLNs) preparation, illustrating both conventional and emerging strategies for the preparation of solid lipid nanoparticles (SLNs). The most common strategies shown on the left-hand side include solvent methods, double emulsion process, homogenization, and microemulsification. The more advanced strategies on the right-hand side are shown, such as membrane contractors, supercritical fluid methods, and ultrasonication. Each step is accompanied by icons of equipment applicable to the method to enhance visualization (Akanda et al. 2023; Nemati et al. 2024). Created with BioRender.com

Solid lipid nanoparticles: advantages, composition, delivery routes, and applications in diabetic wound healing: Solid lipid nanoparticles (SLNs) are one of the aqueous colloidal drug delivery dispersions with rigid biocompatible and biodegradable lipid matrix. SLNs are considered the first nano solid lipid-based carrier replacing the old liposomes and nanoemulsions to overcome their drawbacks. SLNs mainly consist of lipids which are solid at the body or room temperature, these lipids form a matrix in which the active constituents can be attached, dissolved in or adsorbed out of it, in addition to lipids and therapeutic agent there are surfactants added to stabilize the system and to disperse these biophysical lipids, however they can be dispersed in water. SLNs were introduced since 1991 by Gasco and Muller to replace and overcome the drawbacks of liposome as a traditional lipid based drug delivery (Arabestani et al. 2024). Liposomes undergo poor encapsulation, leaking of drug, instability and degradation as they are made of phospholipids. Contradictory, SLNs solved these disadvantages by controlling the release of the drug via being entrapped inside the solid matrix which led to less leakage, more entrapment and higher bioavailability as well as having chemical and physical stability. Moreover, they can deliver pharmaceutical active agents, both aqueous- and lipid-compatible, to specific area by adding some moieties on its surface (Munir et al. 2024). In several studies, SLNs are presented as being advantageous over other NPs forms as they show more effective tumor-growth inhibitions, extended release profiles, and enhanced colloidal stability compared to liposomal and micellar formulations (Dattani et al. 2023). Solid lipid nanocarrier can be delivered orally, parenterally and topically to treat different diseases, practically skin condition. Most used lipids are biodegradable and biocompatible which make SLNs safe to our body, avoid impact of enzymes that modify the drugs, low in drug efflux through different pumps and increase the drug uptake inside the cells to treat intracellular infection (Bayoumi et al. 2024). Additionally, it can encapsulate many different active constituents therefore adding antibiotics, anti-inflammatory drugs and growth factors contributed to fasten the wound healing process, specifically diabetic wounds (Motsoene et al. 2023; Li et al. 2025). One of the key benefits of solid lipid nanoparticles is the versatility in the preparation techniques along with the widespread accessibility of their components (Mehrdadi and Mehrdadi 2024). (Fig. 4) explains the major composition of SLNs drug delivery system. The active pharmaceutical agent (API) is trapped in an inner core composed of solid lipids, such as triglycerides or stearic acid. The outside surface of the SLNs is emulsified by surfactants including phospholipids (e.g., lecithin), polysorbates (e.g., Tween 80), or polymers (such as PVA, poloxamer). These added surfactants improve stability, lower the particle size and decrease the interfacial tension. Sometimes cosurfactant is added if necessary (German-Cortés et al. 2023). (Fig. 5) provides an overview of various techniques employed to fabricate solid lipid nanoparticles (SLNs) and are categorized as primary and non-primary approaches. Simple processes such as hot and cold homogenization, solvent-evaporation processes, microemulsions, and double emulsion processes are under consideration. The figure further mentions other production methods including spray drying, microfluidics, ultrasonication, and supercritical fluid technologies. It also serves as a beneficial tool for researchers, as it enables them to examine and select appropriate manufacturing methods based on efficiency, scalability, and accurate application needs (Akanda et al. 2023; Nemati et al. 2024). The decision to choose the preparation method depends on the availability of equipment, the characteristics of the chosen active constituents, desired particle size, required route of administration, and the method that ensure the successful incorporation of the therapeutic agent. Each method has different degrees of sustainability, for example, the micro-emulsion method does not require solvents or high-pressure equipment, making it a cost-effective and simple option. Additionally, for polar drugs, the double emulsion technique is more suitable for effective encapsulation (German-Cortés et al. 2023).

Recent studies on coated solid lipid nanoparticles in diabetic wound healing and their limitation: Recent research has demonstrated the efficacy of solid lipid nanoparticles (SLNs) in the treatment of chronic wounds by utilizing a variety of active ingredients and innovative production techniques. Table 7 summarizes these findings in terms of incorporated drug, surface coating, dosage form of choice, study model and the key outcomes. Overall, the SLN-based formulations are demonstrated to have a range of therapeutic effects in diabetic wound healing, including enhanced antibacterial activity (Kiymaci et al. 2022), stimulated collagen formation (Fatima et al. 2022), and anti-inflammatory with minimal scarring (Arantes et al. 2020). Additionally, chitosan-coated SLNs were found to achieve more effective control of the wound microenvironment compared to uncoated systems, with the benefits of polymer–lipid interactions (Arantes et al. 2020). Nevertheless, limitations persist, for example variable drug loading, limited stability and incomplete cytotoxicity reporting, and unspecified scalability for manufacture. Therefore, although SLNs are considered a promising delivery vehicle, reproducibility and therapeutic safety will require more future efforts to prioritize standardized protocols, long-term biocompatibility testing, and translation to scalable, clinical-grade production.

**Table 7:** Overview of recent SLN-based approaches in chronic wound healing

Drug/active ingredient	Surface coating	Dosage form	Key findings	References
Fluoxetine	Uncoated	Carbopol gel 1%	Sustained drug release and significantly enhanced the process of wound healing due to higher formation of hydroxyproline level which is responsible for collagen synthesis and tissue regeneration enhancement, in vitro and in vivo	(Fatima et al. 2022)
ATRA	Chitosan	Film	Reduction in leukocytes in wound sites leading to faster wound healing and reducing tissue scars, in vitro and in vivo	(Arantes et al. 2020)
Sesamol	Uncoated	Carbopol gel 1%	Encapsulation significantly increased the antibacterial activity of sesamol by 200% with higher wound healing ability, better histological, biophysical, and biochemical parameters, more targeted delivery and higher skin retention., in vitro and in vivo	(Deol et al. 2024)
Valsartan	Uncoated	HPMC gel 2%	Sustained drug release, enhanced reepithelization, higher anti-inflammatory effect due to increase production of NO, in vitro and in vivo	(El-Salamouni et al. 2021)
MOX	Uncoated	Not mentioned	Higher bacterial uptake up to 57.29% compared to drug solutions, better antibacterial especially for *E. coli* and acceptable cytotoxicity for the prepared SLNs, in vitro and in vivo	(Kiymaci et al. 2022)

*ATRA* all-trans retinoic acid, *MOX* moxifloxacin, *NO* nitric oxide, *TGF-β* transforming growth factor-beta, *MMPs* matrix metalloproteinases, *VEGF* vascular endothelial growth factor

**Fig. 6:**
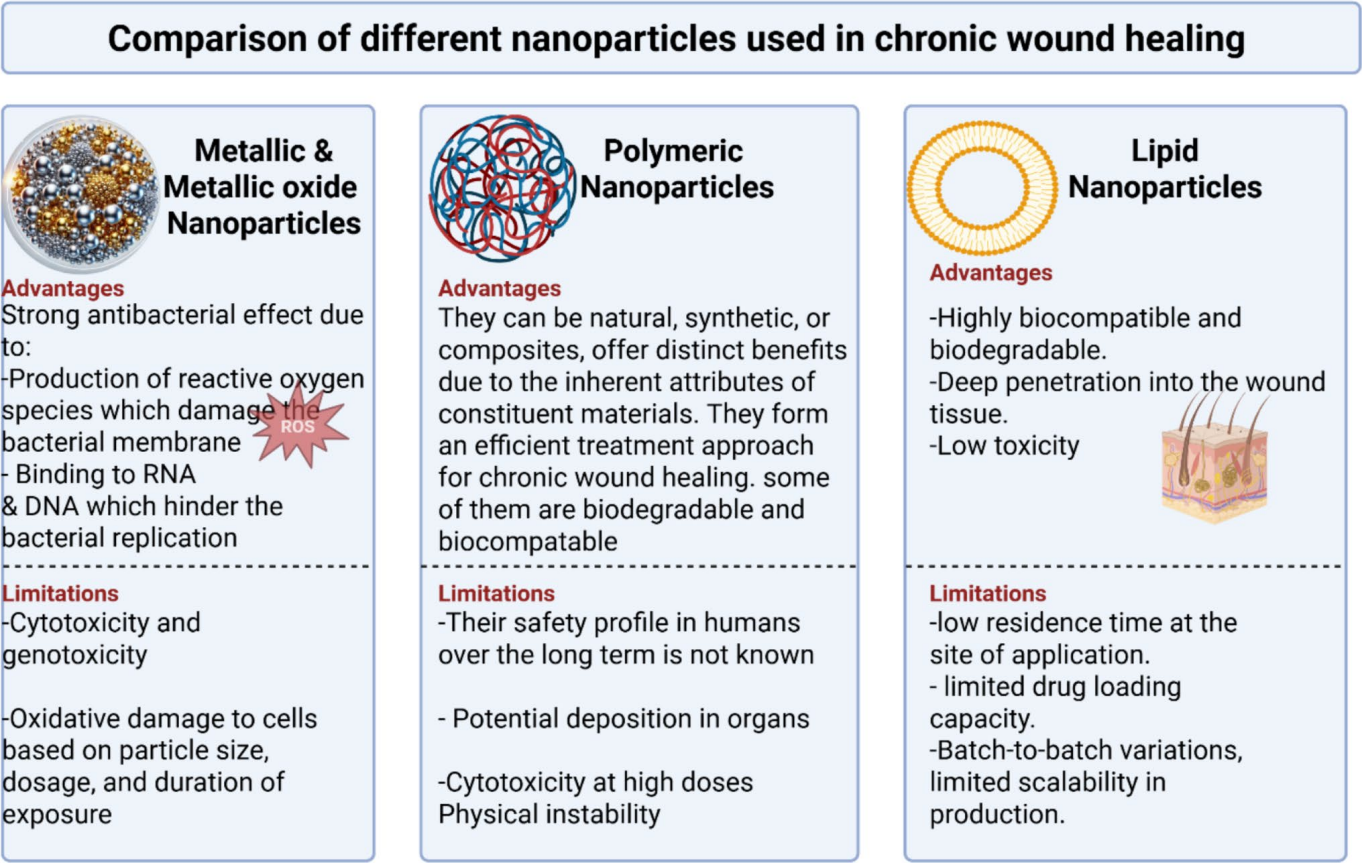
Comparison of the advantages and limitations of different types of nanoparticles for the treatment of chronic wounds. It evaluates the therapeutic potential and translation problems of metallic/metal oxide nanoparticles (Pormohammad et al. 2021; Dikshit et al. 2021; Sheikh-Oleslami et al. 2023; Mahjoubian et al. 2023; Faghani and Azarniya 2024; Medic et al. 2024), polymeric based nanoparticles (Bernal-Chávez et al. 2020; Shalaby et al. 2022; Nunes et al. 2022; Gillella et al. 2024; Fu et al. 2024; Chen et al. 2025), and lipid based nanoparticles (de Souza et al. 2020; Motsoene et al. 2023; Wathoni et al. 2024; Ullah et al. 2024; Gangavarapu et al. 2024; Rehman et al. 2024; Li et al. 2025; Alloush and Demiralp 2025). Created with BioRender.com

Nanostructured lipid nanocarriers: Nanostructured lipid carriers (NLCs) consist of a liquid core (mixture of liquid and solid lipids), an aqueous phase, and surfactants (such as lecithin, poloxamers and Tween 80). The most commonly used solid lipids are stearic acid, palmitic acid, cetyl palmitate, while liquid lipids include capric triglyceride, miglyol 812 or squalene (D’Souza and Shegokar 2020; Houacine et al. 2020). They are advanced lipid-based nanoparticles that possess the capability of enhancing drug stability, improving bioavailability, and allowing controlled drug release, hence making them an important addition to chronic wound healing. Similar to SLNs, their excellent biocompatibility and functionality render them suitable for the encapsulation of several therapeutic molecules. However, unlike SLNs, they mainly incorporate non-polar drug, as the incorporation of polar drugs in the mixture of liquid oil and solid lipids is challenging and leads to significantly low entrapment efficiency percentage (Kasongo et al. 2011). Recent studies in NLCs for the treatment of diabetic wounds as shown in Table 8. Overall, nanoparticles hold great therapeutic promise against diabetes, but all classes possess strengths and limitations that must be carefully assessed. Table 9 provides a side-by-side overview of different nanocarriers (NLCs, SLNs, liposomes, niosomes, and inorganic/ceramic NPs) based on key criteria such as drug loading, release control, toxicity, regulatory maturity, scalability for production, and cost. (Fig. 6) presents a visual comparison of the advantages and limitations of different nanoparticles for the treatment of chronic wounds. Together, Fig. 6 and Table 9 permit a comprehensive assessment of the relative effectiveness and suitability of these nanocarriers for drug applications.

**Table 8:** Detailed analysis of recent advances of nanostructured lipid carriers in managing diabetic wounds

Drug/active ingredient	Surface coating	Dosage form	Key findings	References
Co-loading of Simvastatin and Adenosine	Uncoated	Poloxamer 407 gel 20%	In vivo tests indicate better wound healing, high skin retention, anti-inflammatory effect and improve cell proliferation and migration	(Gomes Daré et al. 2024)
α-tocopherol, quercetin, and Tea tree oil	Sodium alginate or Chitosan	Poloxamer 407 gel 0.5%	In vitro and vivo studies (HET-CAM) showed improvement in the fibroblast migration, reduction in transepidermal water loss and enhancement in the delivery of antioxidants by 74 to 180%	(Costa-Fernandez et al. 2021)
EGF and Curcumin	Uncoated	Gel	Sustained release of drugs, enhanced migration and proliferation of fibroblast and accelerate antioxidant enzyme activity, in vitro and in vivo	(Lee et al. 2020)
rhTM	Uncoated	Carbopol gel	Sustained release kinetics, physical stability and enhanced cell migration of HaCaT, in vitro and in vivo	(Hsueh et al. 2021)
Phenytoin	Uncoated	Hydrogel	Reduction in wound size by 95.82 ± 2.22% and promote the healing process of diabetic foot ulcers without side effects, in vitro and in vivo	(Motawea et al. 2019)

*HET-CAM* Hen’s egg test-chorioallantoic membrane, *EGF* Epidermal Growth Factor, *rhTM* recombinant human thrombomodulin, *HaCaT* = human epidermal keratinocyte cell line

**Table 9:** Comparative evaluation of inorganic nanoparticles, liposomes, niosomes, NLCs, and SLNs on the drug loading and encapsulation efficiency, release control, toxicity, regulatory maturity, scalability and cost

Parameter	SLNs	NLCs	Liposomes	Niosomes	Inorganic/ceramic NPs
Drug loading/Entrapment efficiency (EE%)	**Loading:** ~ 5–15% w/w **EE:** Vary from 30–90% according to the drug incorporated & preparation method (Duan et al. 2020; Pandey et al. 2021)	**Loading:** relatively higher than SLNs ~ 5–30% due to the liquid portion of the lipids **EE:** higher encapsulation in case hydrophobic drugs (Chauhan et al. 2020)	**EE:** variable; **50–95%** high in lipophilic drug unless advanced remote-loading techniques used such as Doxil drug formulation (Liu et al. 2022)	**Loading/EE:** often **5–17%**;/ ≈ **40–80%** depending on the surfactant and hydration (Moammeri et al. 2023)	Mainly acts as active ingredient rather than carriers. Loading differs depending on ion release mechanism or surface-adsorbed drug. With minimal EE% in ceramics (Unnikrishnan et al. 2023)
Release control	Sustained, matrix diffusion controlled release over 24 -72 h. (Pandey et al. 2021)	Controlled- matrix diffusion and erosion release with reduced burst release (extends over days) (Saedi et al. 2018; Haider et al. 2020)	Fast release unless PEGylation, multilamellar can extend release to days. (Liu et al. 2022)	Moderate controlled release based on surfactant and lamellarity (Mawazi et al. 2025)	Rapid effect in hours, due to ion release or ROS-mediated activity; functionalized carriers can slower the release (days). (Unnikrishnan et al. 2023)
Toxicity concerns	Low toxicity due to high biocompatibility, biodegradability (Yaşayan et al. 2023) however the toxicity vary according to the surfactant used (Ghasemiyeh and Mohammadi-Samani 2018)	Low–moderate depended on the type of liquid lipid and surfactant (Elmowafy and Al-Sanea 2021)	Low–moderate due to the biocompatibility of phospholipids, though they are susceptible to oxidative degradation (Peng et al. 2022)	Moderate toxicity mainly influenced by the type and concentration of surfactant (Yaghoobian et al. 2020)	High toxicity risk as they are inherently cytotoxic due to ion release and ROS-generation therefore the toxicity is highly dependent on particle size, concentration, and exposure time (Bhatti et al. 2022)
Regulator-y maturity	Emerging; numerous topical pre-clinical and early clinical exist with small numbers of SLNs have completed the full market approval (Gugleva and Andonova 2023)	Preclinical literature is growing and evolving, but there are few human products currently on the market (Patil et al. 2023)	High-level regulatory maturity: guided by the existence of explicit standards like the FDA’s release of Liposome Drug Products, a number of liposomal products have been approved for topical and systemic applications. (Wang and Grainger 2022)	low to middle-level regulatory maturity: niosomal products have far fewer regulatory precedents and approved drugs than liposomes, and they are largely in preclinical or early clinical development. (Moammeri et al. 2023; Teron et al. 2024)	Partially approved: Although there are some silver-based wound dressings approved for use, the nanoformulations are regulated and require careful toxicological assessment.(Ma et al. 2024)
Manufacturing & scale-up feasibility	Moderate scalability: Established processes such as microemulsions and hot/cold homogenization are well established. Nonetheless, particle size control during lyophilization, maintenance of polymorphism, and batch-to-batch uniformity remain challenging issues (Alfutaimani et al. 2024)	Moderate to problematic scalability: Repeatability and scaling are hindered by mixing liquid and solid lipids, making processes more complex (Gomaa et al. 2022)	Moderate to high scalability: Commercial production can be conducted, but sterilization and Chemistry, Manufacturing, and Controls (CMC) regulation compliance are expensive (Chelliah et al. 2025)	Moderate scalability: There are simpler methods of preparation, but reproducible scale-up and surfactant source must be qualified (Moammeri et al. 2023)	Variable scalability: Physical synthesis methods are scalable, but waste management, toxicology assessment, and Good Manufacturing Practice (GMP) are added in complexity and cost (Kaymaz et al. 2023)
Cost of manufactu-ring	Low to moderate material expense, but overall expense can rise with process controls like lyophilization and stability tests (Stahl et al. 2024)	Moderate expense: form complexity in the sense of the addition of both liquid and solid lipids can provide a moderate price (Nguyen et al. 2022)	Moderate to high expense: existing markets for some products are willing to accept higher expense, yet use of phospholipid and sterilization elevate manufacturing expense (Chelliah et al. 2025)	Low to moderate cost: QC charges are implemented but surfactants are comparatively less costly than phospholipids (Antonara et al. 2025)	Nanoparticles of monometallics are cheap in raw material, but downstream safety and regulation costs can be high (Percoco et al. 2025)

### Strategies of nanoparticles-based systems for biofilm eradication in chronic wound healing

Despite remarkable progress achieved using different nanosystems, persistent microbial colonization and biofilm development still impede full healing of diabetic wound. Biofilm-expressing pathogens such as Staphylococcus aureus and Pseudomonas aeruginosa, are frequently detected on diabetic chronic wounds, where they hinder antimicrobial penetration, promote antibiotic tolerance, and inhibit tissue repair. Consequently, antibiofilm-based devices that integrate physical, chemical, and biological mechanisms for eradicating recalcitrant infections and triggering tissue regeneration are gaining prominence in modern nanotherapeutic manifestations. Nanoparticle platforms are well poised to break these barriers by synergizing physico-chemical disruption, signal interference, quorum-sensing interference, and enzymatic/oxidative matrix degradation (Jing et al. 2024), as detailed below.

#### Charge design-electrostatic disruption

Besides the advantages of coating the NPs with chitosan as mentioned previously. It also facilitates efficient attachment to the negatively charged bacterial cell envelope, thereby activating membrane perturbation, increasing local NPs concentration, and enhancing antimicrobial activity (Jing et al. 2024). As mentioned in one study (Davidson et al. 2023), chitosan-derived nanovesicles enhanced fibroblast migration and suppressed Pseudomonas aeruginosa biofilm both in vitro and in vivo. In addition, compared to unmodified chitosan, the quaternized chitosan derivatives more effectively inhibited Staphylococcus aureus biofilm formation, emphasizing the importance of increasing charge density (Miranda et al. 2025). A systematic analysis of the mechanisms for NPs disruption of the biofilms indicates that electrostatic interactions contribute significantly to NPs penetration into biofilms and to improved wound-healing outcomes (Sedighi et al. 2024).

#### Quorum-sensing description- maturation inhibition

In addition to the previous mechanism of biofilm inhibition, it was reported that bacterial chemical signaling that regulates biofilm maturation and virulence-factor expression is also disrupted by quorum-sensing inhibitors (QSIs) and quorum-quenching (QQ) enzymes. Nanocarrier-based delivery of QSIs are found to elevate local concentrations, stabilizes labile inhibitors, and allows controlled release at the wound site (Blanco-Cabra et al. 2024; D’Aquila et al. 2024). For example, halogenated furanones-small-molecule acyl homoserine lactone -type signaling inhibitors are loaded onto polymeric aerogels, and other nanoplatforms achieving > 90% inhibition of Pseudomonas aeruginosa biofilm biomass in vitro, and a substantial reduction in biofilm burden in wound models (Proctor et al. 2023). Moreover, enzyme-based nanoparticles with QS mechanisms provide enzyme stability and enable sustained hydrolysis of signaling molecules. Recent findings also indicated that enzyme-loaded nanocarriers can downregulate expression of virulent genes in the respective pathogens and make biofilms antibiotic-sensitive (Sompiyachoke and Elias 2024). Furthermore, inorganic nanoparticles have been reported to disrupt quorum-sensing proteins and diminish signaling-dependent behavior in vitro and in silico which represent a further QS-suppressing mechanism (Varma et al. 2023; Kanak et al. 2023).

#### Enzyme/oxidant co-therapies

Another successful approach is the co-delivery, via nanoparticle carriers, of matrix-degrading enzymes (e.g., DNase I, Dispersin B, proteases) or regulated oxidant donors (most notably nitric oxide; NO) that enhance biofilm degradation, facilitate immune clearance and restore antibiotic susceptibility (Li et al. 2023). For example, DNase I degrades extracellular DNA (eDNA) in established biofilms which administered with silver-sulfadiazine–solid lipid nanoparticles (SSD-SLNs) or from lipid/gel matrices that significantly reduces biomass, and improves wound closure in infected models; complete healing was observed in a 21-day rat model (Patel et al. 2019). Furthermore, several diabetic wound models in vivo have shown diminished bacterial load and improved granulation with NO-donor nanoparticles (Roberts et al. 2024). NO-releasing nanocarriers provide a sustained local flux of NO that disperses biofilms with the delivery of immediate antibacterial efficacy (Nafee et al. 2024). Dual-mode therapies combining enzymatic cleavage and redox action (e.g., enzyme-stabilized lipid or polymer nanoparticles co-delivering NO donors) surpass single-mode therapies, providing synergistic degradation of extracellular polymeric substance and enhanced re-epithelialization (Tortella Fuentes et al. 2024).

#### Hybrid and nanocomposite approaches: a translational perspective with synergistic multimodal outcomes

To achieve synergistic antibiofilm and healing outcomes, hybrid nanoparticle constructs such as metal–polymer composites or NPs-loaded hydrogels combine multiple antibacterial modalities, including charge-mediated disruption, controlled release via lipid carriers, and wound-bed adherence provided by hydrogel scaffolds. (Table 10) provides an overview of such multimodal system examples. Recent investigations in diabetic wound models have shown that chitosan–silver nanocomposite hydrogels and multifunctional Ag/NO co-loaded dressings can eradicate established Pseudomonas aeruginosa and Staphylococcus aureus biofilms, while enhancing collagen deposition and re-epithelialization (Alven and Aderibigbe 2024; Xie et al. 2024). Additionally, SLN-based hydrogel systems and polymer–metal oxide nanohybrids exhibit improved retention at the wound bed, prolonged antimicrobial activity, and biocompatible degradation (Pammi et al. 2025). For clinical translation, standardized and measurable endpoints such as biomass or CFU-based biofilm reductions, downregulation of quorum-sensing markers, matrix degradation assays (eDNA and polysaccharides), in vivo wound-closure kinetics, and comprehensive safety assessments (cytotoxicity, inflammation, systemic biodistribution), all are essential for NPs-based antibiofilm strategies (Chegini et al. 2025). It is worth mentioning that to facilitate regulatory evaluation and scale-up production; mechanistic assays of DNase activity, release kinetics and zeta potential should be correlated with corresponding biological outcomes such as biofilm disruption, wound-closure rate, and histology findings. Establish such A robust, evidence-based evaluation framework will help nanocomposite wound therapies progress from preclinical proof-of-concept to clinical use (Sedighi et al. 2024).

**Table 10:** Concise overview of representative multimodal nanoparticle systems and their antibiofilm mechanisms

Multimodal nanoparticle systems	Mechanisms	Model	Key performance endpoints
Natural-product quorum sensing inhibitors (e.g. Allicin, curcumin, eugenol, furanones, flavonoids) + NPs	1-Quorum-sensing disruption: inhibit signal synthesis, suppresses QS-regulated gene expression	• In vitro QS and biofilm models	• Reduction of QS signal production • Significant bio‑film inhibition of P. aeruginosa and S. aureus (Ugo et al. 2025)
Chitosan + NO donor, AgNPs + ciprofloxacin hydrogel	1-Charge‑design/electrostatic disruption;Ag⁺ released 2-. Enzyme/oxidant co‑therapy; NO release	• In vitro antibacterial assays (MIC) & biofilm inhibition • In vivo infected wound healing	• ~ 65% bio‑film inhibition of P. aeruginosa • ~ 30% wound closure by day 7 and ~ 90% by day 11 (Chegini et al. 2025)
Chitosan + SLNs + silver sulfadiazine + DNase-I	1-Charge‑design/electrostatic disruption; Ag⁺ released 2- Enzyme/oxidant co‑therapy; DNase‑I release	• In vitro antibacterial assays (MIC) & biofilm inhibition • In vivo burn-wound healing	• 96.8% bio‑film inhibition of P. aeruginosa • 100% wound closure at 21 days • cytocompatibility 70% fibroblast viability (Patel et al. 2019)
Ag/Fe NPs + NO	1-Charge‑design/electrostatic disruption; metal ion released 2-.Enzyme/oxidant co‑therapy; NO release	• In vitro antibacterial assays (MIC) &biofilm inhibition (CFU count) • In vivo infected wound healing	• > 70% bio‑film inhibition • significant acceleration of wound closure (Padaga et al. 2025)
ZnO NPs + Bacterial cellulose + borax hydrogel	1-Charge‑design/electrostatic disruption; metal ion released 2-Enzyme/oxidant co‑therapy;	• In vitro (biofilm inhibition)	• ~ 65.5% bio‑film inhibition of S. aureus • ~ 71.7% bio‑film inhibition of P. aeruginosa (Kart et al. 2024)

*NPs* nanoparticles, *AgNPs* silver nanoparticles, *Ag⁺* silver ions, *NO* nitric oxide, *SLNs* solid lipid nanoparticles, *DNase I* deoxyribonuclease I, *ZnO NPs* zinc oxide nanoparticles, *Fe NPs* iron nanoparticles, *CFU* colony-forming units, *QS* quorum sensing, *MIC* minimum inhibitory concentration

## Advancement from traditional to nanoparticle-driven dosage forms for the healing of diabetic chronic wounds: focus on Thermoresponsive smart gel

**Fig. 7:**
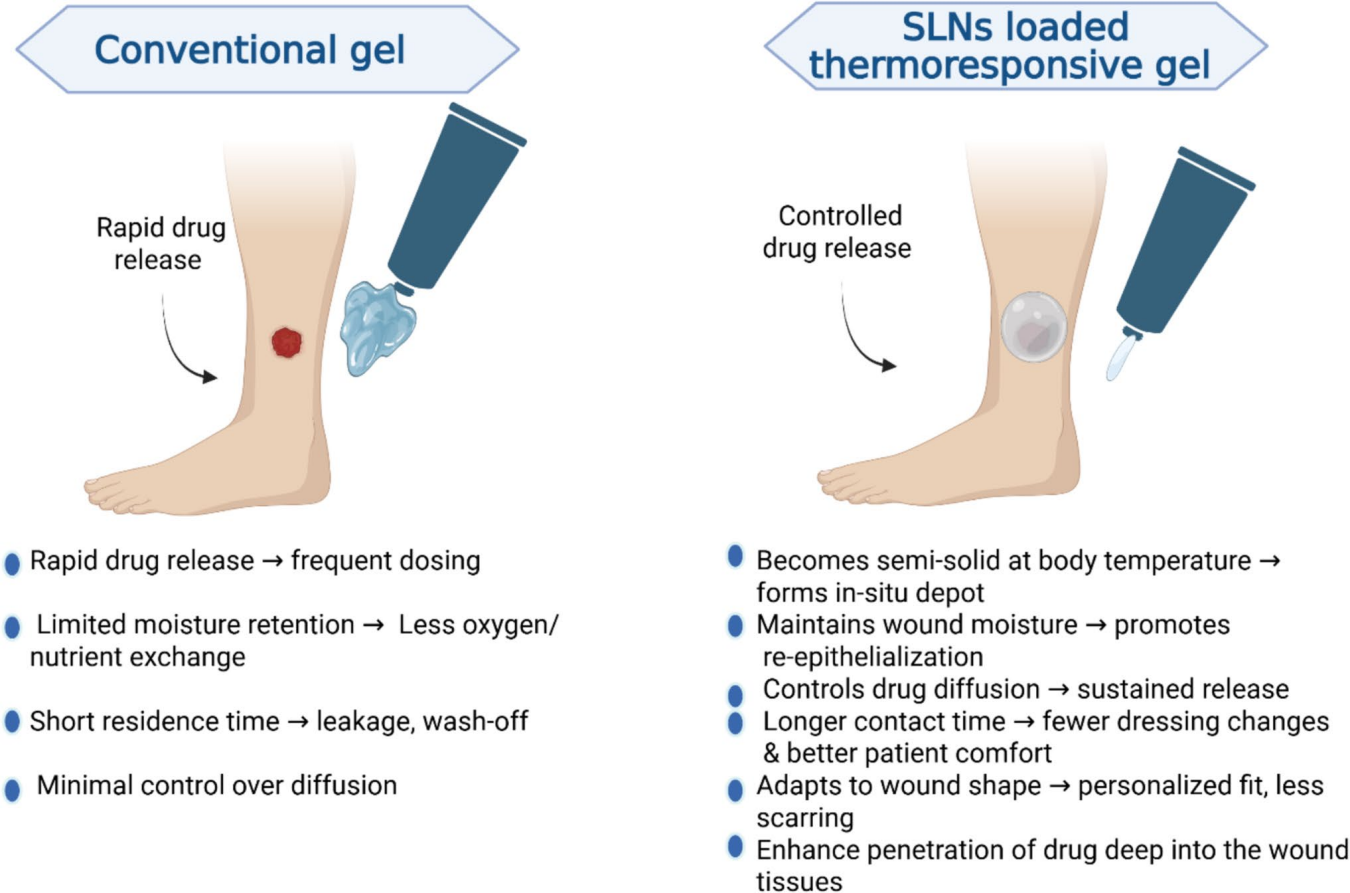
A comparison between SLNs-loaded thermosensitive gels and conventional gels for diabetic wound healing. Traditional gels, with their fast release of drugs, low water holding capacity, short residence time, and absence of diffusion control, necessitate frequent application and may impede the healing of the wound (Wang et al. 2022c). On the other hand, SLN-loaded thermoresponsive gels also undergo a sol–gel transition at body temperature and form an in situ depot that has better moisture retention (Ding et al. 2025)., controlled release, longer residence time, and higher penetration of the drug (Yang et al. 2024). Created with BioRender.com

Recent advances in the management of chronic wounds have led to alternative dosage forms focused on nanoparticles with the aim to enhance therapeutic potency. In the present review, a range of traditional and recent dosage forms including hydrogels, gels, films, nanofiber scaffolds, and wound dressings have been reviewed. For example, bio-adhesive film dosage form provide localized drug delivery along with mechanical support (Pansara et al. 2020). Furthermore, dressings and nanofiber scaffolds with metallic nanoparticles such as gold or zinc oxide are two-way contributing factors to tissue regeneration and preventing infection (Khan et al. 2021; Meng et al. 2024). Based on the highly porous extracellular-matrix–like structure supporting collagen synthesis, encouraging angiogenesis and cell attachment, and facilitating sustained delivery of bioactive agents, nanofibrous wound dressings have found their place as multifunctional scaffolds. In diabetic mice models, full thickness wounds are greatly accelerated in closure by the dressings when they are filled with regenerative as well as antibacterial therapies (Koupai et al. 2025). Moreover, their versatility, biocompatibility, and compatibility with wound physiology. Gels and hydrogels remain one of the most popular forms in wound care. For example, carboxymethyl cellulose/poly (vinyl alcohol) are widely utilized because they can maintain tissue hydration, have prolonged release, and enhance antibacterial activity (Capanema et al. 2023). For gels especially the smart thermoresponsive systems, it was reported that they enhance drug penetration with ease of application (Arafa et al. 2023; Yang et al. 2024). They are particularly beneficial in the treatment of chronic wounds as they maintain moisture within the surrounding tissue for extended periods while maintaining the diffusion of oxygen and nutrients, hence improving the healing process, promoting re-epithelialization, and preventing tissue desiccation. Moreover, they can minimize scarring and provide a personalized fit to the wound site by adapting to various sizes and shapes of defects (Yang et al. 2024). Besides their therapeutic uses, they also help in addressing some patient-related problems such as usability and compliance. They can be delivered directly as low-viscosity fluids that can spread uniformly on irregular surfaces of wounds due to their *in-situ* gelation characteristic. On gelation, they result in a semi-solid depot that is resistant to leakage and stays in contact with the wound for an extended duration of time. This advantage simplifies home wound care, reduces patient discomfort, and decreases dressing procedure frequency. Additionally, the non-irritating, gentle gel matrix enhances comfort during application and removal, thereby enhancing compliance with extended treatment courses. The gel forms upon contact with the wound surface because of the presence of specific polymers known as stimuli-responsive or smart polymers. Theses macromolecules can alter their physical and chemical structures in response to environmental stimuli such as UV light, pH, or temperature. Thermoresponsive polymers can be classified into two broad categories: those exhibiting an upper critical solution temperature (UCST) and those exhibiting a lower critical solution temperature (LCST). LCST polymers become less soluble and gel above a certain temperature (phase transition at ~ 32 °C), because of a hydrophilic-to-hydrophobic phase transition that promotes chain association and gelation. Their ability to remain liquid at room temperature for easy application and solidify rapidly on entry into the body temperature environment to create a local drug depot delivering prolonged release and sustaining the moist healing environment is particularly useful in wound care (Ding et al. 2025). UCST polymers, by contrast, gel on cooling below a lower critical temperature, usually through coil-to-helix transitions or hydrogen bonding (such as in agarose-based systems). UCST systems find their use favorably in stable dressings and cold-induced material shaping, if less commonly in applications on the skin (Xin et al. 2025). An understanding of these processes informs the selection and design of thermos-responsive carriers suitable for specific applications and the temperature condition at the wound site. Poloxamers are representative examples of that exhibit LCST with a wide range of applications in topical and transdermal drug delivery systems. They can turn into semi-solid when exposed to body temperature (Rad et al. 2024) usually at 33–37 °C, thus being suitable for dermal wound therapy (Bejenaru et al. 2025). They are also fit to be formulated as a binary system of Poloxamer 407/188 for drug delivery that has been demonstrated reproducibly in vitro gelation and prolonged release (Ruan et al. 2022), particularly at a 21:9 (w/w) ratio (Arafa et al. 2023). This ratio forms gel at around 36–37 °C, allowing the formulation to be liquid at room temperature and become semisolid at the desired skin temperature. The composition is rationale in purpose: Poloxamer 188, with greater hydrophilicity (PEO content), gives softness to the gel matrix, improving spreadability and elasticity, while Poloxamer 407, which is thermogelling and more hydrophobic, gives structural rigidity and ensures rapid sol–gel transition (Chen et al. 2021). Thermoresponsive systems have also made use of different polymers. For example, Pluronic F127 is extensively utilized due to its capability for thermoreversible gelation. It has been combined with other polymers, such as alginate (20% w/v PF127 with 1% w/v alginate) (Cao et al. 2020) that could be made through the cold approach, and HPMC (1 g with 15 g PF127) (Arafa et al. 2018), in order to confer strength and tunability. These polymer blends are superior to poloxamer-alone systems in sustained drug release and wound healing due to improved mechanical strength, viscosity, and better control over gelation temperature. Drug release kinetics from the gel matrix is predominantly diffusion-controlled by Higuchi-type diffusion. These findings agree with the physicochemical characteristics of the formulations, which are typically described by matrix diffusion behavior (Fatima et al. 2022). Recent studies enumerate several innovative thermoresponsive gel such as: sprayable hydrogels allow for application on irregular wound surfaces (Gu et al. 2023); thermoresponsive gels containing lidocaine provide extended analgesia with more contact time on oral wounds, which encourages patient comfort and compliance (Supachawaroj et al. 2024); in addition to self-healing hydrogels that reduce dressing disturbances and handling convenience (Wang et al. 2022c). Another example is the inclusion of platelet lysate-loaded PLGA nanoparticles in thermoresponsive matrices (Supachawaroj et al. 2025), likewise, injectable and bioactive gel demonstrated that longer residence-time cross-linked at the wound site could augment tissue integration, inhibition of infection, and enhanced re-epithelialization (Shahriari-Khalaji et al. 2025). Overall, Incorporation of SLNs into these smart gel matrices further enhances control over diffusion processes (Jenning et al. 2000). The development from conventional gel formulations to nanoparticle-initiated, thermoresponsive smart gels for advanced chronic wound healing is illustrated in (Fig. 7). Furthermore, a comparative overview of different recent thermoresponsive formulations is summarized in (Table 11).

**Table 11:** Comparative overview of different thermos-responsive gel formulations

Polymer base	Drug	Polymer concentration (% w/v)	Gelation temp (°C)	Rheological behavior (G′/G″)	Drug release rate	Batch to batch reproducibility	Key test methods	References
Pluronic F127 + Alginate	GSNO	20: 1 w/v% PL/AL	PL alone = 26.4 ± 0.2 °C; PL/AL = 24.2 ± 0.3 °C; GSNO-loaded gel = 23.4 ± 0.2 °C	the sol–gel transition near 20–24 °C (rapid G′ increase), and a solid-like gel at 37 °C	an initial burst (≈ 8% released in 4 h, 29% in 12 h) followed by sustained release for 7 days t_1/2_ of ≈ 24.5 h	< 5% variation	Gelation temperature (tube inversion method), rheometer, DSC, in-vitro release cytotoxicity, antibacterial efficacy & in vivo in MRPA infected mice	(Cao et al. 2020)
Pluronic F 127 + PEG 400	HLRE & PPE	Pluronic F 127 20–25% w/v + 22% PEG 400	at $25.7\pm 0.3$ to $26.7\pm {1.2}^{\circ }{\mathrm{C}}$ of optimized gels	$0.45\pm 0.53$ N = hardness valve indicating (G′ > G″)	PPE: limited antibacterial activity, HLR: high activity + rapid wound closure (74% ± 12% to 89% ± 2% within 24 h)	n/a	Rheology analysis, in vitro wound-scratch (cell migration) assay, physicochemical tests, and antibacterial testing of PPE	(Faris Taufeq et al. 2023)
Poloxamer 407/188 (21: 9 w/w)	CIP	30% w/w (P407 + P188)	Nanoparticle-loaded gels at 36.9 ± 0.3 °C, while the free-CIP gel at 36.0 ± 0.4 °C	Gels with pseudoplastic, shear-thinning flow; viscosity drops from ~ 15 000 cP to ~ 3 100 cP	Release after 72 h is 50.03 ± 0.73% & 77.98 ± 3.12%; follows Higuchi kinetics with non‑Fickian diffusion	Significant consistency across batches (p < 0.001)	Gelation temperature; rheology by Brookfield viscometry; in-vitro release; antibacterial efficacy	**(**Arafa et al. 2023**)**
Poloxam-er 407	Diclofenac sodium	17% and 20% (w/v)	17% gels at 27.7 °C and 20% gels at 23.0 °C Gels with 1% diclofenac gelled at 23–30 °C	The higher the poloxamer the higher the Storage modulus (G′)	Sustained over 24 h; zero-order kinetics	Triplicate trials verified the formulation’s uniformity	Rheology, temperature ranges, in vitro release, Ex vivo permeation	(Russo et al. 2020)
Poloxamer 407	Polyhexanide	18–22% (w/v)	~ 33–36 °C	G″ > G′ occurs above 33 °C	Diffusion- controlled release over 24 h	Triplicate formulations with < 5% variation	Rhinometry, in vitro release, antimicrobial test and cytocompatibility	(Alparslan et al. 2025)

*PL/AL* pluronic F127 with natural alginate, *GSNO* S‑nitrosoglutathione, *(G′/G″)* G″ is the loss modulus indicative liquid-like response, whereas the storage modulus G′ is indicative of solid-like response. When G′ is greater than G″ (G′ > G″), the sol–gel transition temperature is reached; *DSC* differential scanning calorimetry, *MRPA* Methicillin-Resistant *Pseudomonas aeruginosa*, *PEG* polyethylene glycol, *HLRE* Hot-water extracts of *Lignosus rhinoceroses*, *PPE Punica granatum* peel, n/a not applicable, *CIP* ciprofloxacin hydrochloride

## Obstacles and regulatory consideration in clinical translation of nanoparticles driven wound treatment

**Fig. 8:**
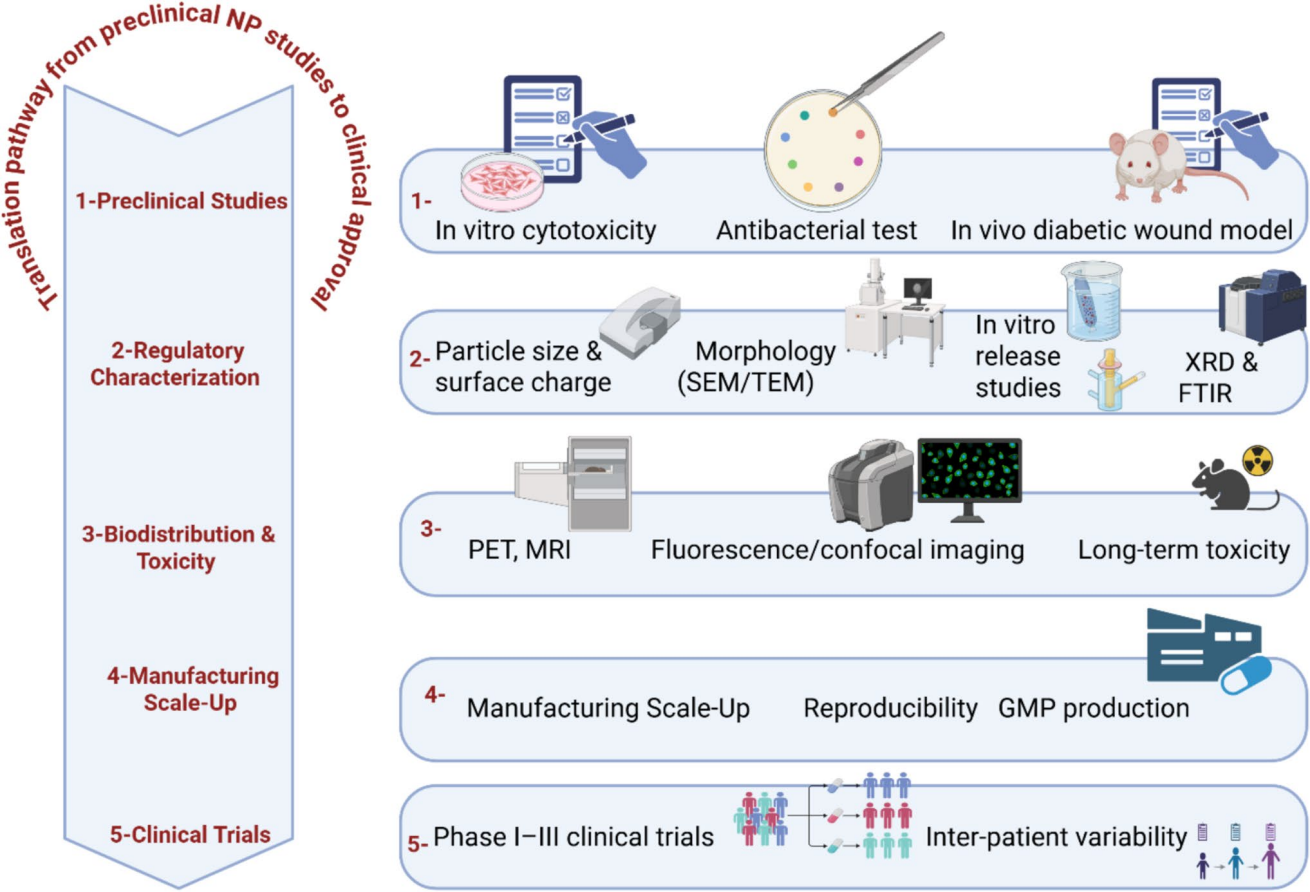
Translation pathway of how nanoparticle studies in the preclinical phase proceed to clinical approval. The workflow includes: Preclinical characterization: in vitro cytotoxicity tests, antibacterial activity measurements, and in vivo diabetic wound models; Regulatory characterization: establishing particle size, surface charge, morphology, and release profiles (Rodríguez-Gómez et al. 2025); Biodistribution and toxicity assessments: utilizing modalities such as positron emission tomography (PET), magnetic resonance imaging (MRI), fluorescence imaging (Kumarasamy et al. 2024), and long-term toxicity assessment (Foulkes et al. 2020; Domb et al. 2021); scale-up to production: with emphasis on reproducibility and GMP-compatible production (Zeng et al. 2022). Clinical trials: Phase I–III trials with assessment of inter-patient variation and therapeutic response. Created with BioRender.com

Despite promising preclinical results, several obstacles still hinder clinical translation of nanoparticle-based wound therapies. Regulatory requirements are stringent: rather than being informed by nanotechnology-specific guidelines, developers must navigate a complex FDA framework that addresses each product on an individual basis. Submissions must adhere to ISO/ASTM and Nanotechnology Characterization Laboratory (NCL) standards and include validated analytical methods, such as electron microscopy and dynamic light scattering (DLS) (Đorđević et al. 2021), thorough nanoparticle characterization is required. This includes comprehensive reporting of physicochemical properties like morphology, particle size distribution, surface charge, drug loading, and release kinetics (Rodríguez-Gómez et al. 2025); batch-to-batch size uniformity within predetermined parameters (Csóka et al. 2021) and accelerated clearance profiling. To establish dosing regimens, biodistribution and in vivo tracking studies are also required. Advanced imaging techniques such as PET, MRI, and fluorescence-based strategies enable monitoring of systemic clearance, tissue retention, and time distribution (Kumarasamy et al. 2024). Safety concerns remain unresolved as nanoparticles may cause immunological activation, oxidative stress, and chronic toxicity, and potentially accumulate in liver, kidney, and spleen, where long-term toxicokinetic profiling is a critical issue (Foulkes et al. 2020; Domb et al. 2021). Mass production of lipid nanoparticles remains technically challenging, with tedious processes such as lyophilization rendering it costly (Zeng et al. 2022). To address these obstacles, a systematic approach is proposed, as presented in (Fig. 8). The process advances step by step from characterization and biodistribution studies, through toxicity assessment and scale-up production, to compliance with regulations.

## Critical perspective and conclusions

Diabetic chronic wounds and particularly diabetic foot ulcers continue to pose a significant therapeutic challenge due to their high risk of severe complications, compromised healing, and intractable infection. Standard treatments tend to fail due to systemic side effects, limited bioavailability, and inadequate drug delivery. This crucial comparative review of nanotechnology-based methods highlights the privileged position of solid lipid nanoparticles (SLNs) as the therapeutic treatment of chronic diabetic ulcers. Moreover, surface modification with biopolymers such as chitosan and hyaluronic acid offers a dual advantage by enhancing cellular uptake and antimicrobial activity and promoting wound-specific biological processes. The incorporation of SLNs into thermosensitive gel systems represents a paradigm in wound therapy; these smart gels react to wound microenvironmental conditions, minimizing drug loss, enabling sustained release, and conferring adhesion to the irregular shapes of wounds. However, regulatory barriers, biofilm penetration difficulties, and batch reproducibility concerns continue to hinder clinical translation. Based on the recent advances in wound treatment with nanoparticles, future research should focus not just on developing standardized procedures and environmentally friendly, scale-up synthesis methods but also on optimizing formulations with care for maximizing drug loading, surface-coating durability, reproducibility, and controlled release. In addition, efforts should also be directed toward clinical assessment, conducting early-stage clinical trials done to evaluate infection control, rates of wound closure, and safety among diabetic wound patients. On these grounds, surface-functionalized SLNs, such as those in the process of being chitosan-coated or conjugated with metal or antimicrobial agents, offer a strategic approach to disrupt biofilm matrices, promote antibiotic penetration, and improve therapeutic performance. Combinatorially, treatment systems compromising functionalized SLNs loaded with drugs in thermoresponsive gels represent a leading frontier in diabetic wound care. They not only serve as delivery vehicles but also provide structural support and modulate physiological behavior, even more than conventional dressings and non-nanostructured hydrogels.
